# Neural Network-Based Adaptive Attitude Controller for a Micro-Unmanned Aerial Vehicle

**DOI:** 10.3390/s26216797

**Published:** 2026-10-27

**Authors:** Gennaro Raspaolo, Salvatore Rosario Bassolillo, Luciano Blasi, Egidio D’Amato, Immacolata Notaro

**Affiliations:** 1 Department of Engineering, Universitá degli Studi della Campania “L. Vanvitelli”, Via Roma, 29, 81031 Aversa, CE, Italy; gennaro.raspaolo@unicampania.it (G.R.); salvatore.bassolillo@gmail.com (S.R.B.); luciano.blasi@unicampania.it (L.B.); immacolata.notaro@unicampania.it (I.N.); 2 Department of Science and Technology, Universitá degli Studi di Napoli “Parthenope”, Centro Direzionale di Napoli, Isola C4, 80143 Napoli, NA, Italy

**Keywords:** attitude control, robust control, disturbance rejection, micro quadcopter, neural networks

## Abstract

The deployment of micro-Unmanned Aerial Vehicles (UAVs) for civil applications has exponentially grown in the last decades due to their versatility, low cost and ease of use. However, their effective implementation requires an efficient attitude control system that ensures the tracking of a reference signal, even in the presence of uncertainties or external disturbances. The main aim of this paper is to present the design of a neural-network-augmented $H_{\infty }$ controller for a micro-UAV, to compensate for dynamic uncertainties and environmental perturbations. The effectiveness of the proposed architecture is validated through both numerical sensitivity analyses and experimental tests on a Crazyflie 2.1+ quadrotor mounted on a tilting test bench.

## 1. Introduction

Over the last few decades, UAVs have gained increasing attention due to their versatility and ease of use, both in civil and military applications. Their widespread adoption is mainly due to cost reductions and improvements in low-cost electronics, offering competitive products for a wide range of applications [1,2], including localization and mapping [3,4,5], environmental monitoring [6,7,8] and precision agriculture [9,10,11]. Multirotor drones have shown significant advantages over fixed-wing aircraft in hazardous environments [12,13,14] due to their Vertical Take-Off and Landing (VTOL) capability, maneuverability in confined spaces and the ability to hover over a specific target.

However, multirotor vehicles need a reliable and effective attitude controller to perform precise maneuvers, capable of ensuring vehicle stability even in adverse operating conditions, such as in the presence of wind gusts or disturbances due to unbalanced payloads [15].

Due to their straightforward implementation and ease of tuning, Proportional Integral Derivative (PID) controllers are widely considered as the baseline control architecture for both linear and nonlinear quadrotor dynamics [16,17,18,19,20,21,22,23,24].

The authors of [25] proposed a systematic model-based approach to the design of a UAV research platform, adopting a conventional PID controller for attitude stabilization purposes. A cascaded attitude control strategy is introduced in [26], featuring a PID-based inner loop for angular velocity and a Proportional Integral (PI)-based outer loop for attitude regulation. However, these controllers exhibit poor performance in the presence of system uncertainties or strong external disturbances.

To address performance limitations of classical PID-based strategies, several alternative control architectures have been proposed in the literature. For instance, in [27], a state feedback controller based on the Linear Quadratic Regulator (LQR) paradigm was presented. An adaptive PID controller using sliding mode control approaches was proposed in [28]. To address the nonlinear nature of quadrotor dynamics, several nonlinear control methodologies have been developed. Backstepping control has emerged as an effective solution due to its recursive design procedure and suitability for cascaded systems. The authors of [29] proposed a robust backstepping controller enhanced by disturbance estimation techniques to improve attitude-tracking performance under uncertainties. Another popular nonlinear approach is sliding mode control (SMC), which is known for its robustness against model uncertainties and external disturbances. The application of SMC to quadrotor attitude regulation is investigated in [30], comparing its performance with other nonlinear control strategies.

More recently, optimal predictive and robust control techniques have gained increasing attention. model predictive control (MPC) explicitly incorporates system constraints and optimizes the control action over a finite prediction horizon. A multi-model predictive control framework for quadrotor attitude regulation is introduced in [31], achieving performance close to nonlinear MPC while significantly reducing computational load.

While these approaches can significantly improve tracking performance, their capabilities strongly rely on the availability of accurate models or extensive training data, and they generally do not provide explicit robustness guarantees against uncertainties or persistent disturbances. Furthermore, model-based nonlinear techniques, such as dynamic inversion, are highly sensitive to modeling errors, which may degrade controller performance in real-world operational scenarios [32,33].

In contrast, the robust $H_{\infty }$ control technique has proven to guarantee closed-loop stability in the presence of bounded disturbances and model uncertainties [34,35]. Over the years, several researchers have adopted this kind of technique in UAV applications. The authors of [36] proposed a technique that utilizes feedback linearization for model nonlinearity, while a μ-synthesis controller is used to achieve robust performance in the presence of model uncertainties and external disturbances. The paper presented in [37] proposes the application of an $H_{\infty }$ controller with static gains for the stabilization of an HAPD (High-Altitude Performance Demonstrator) with redundant control surfaces. To effectively stabilize a quadrotor against atmospheric disturbances during flight operations, the authors of [38] used the $H_{\infty }$ paradigm to nullify the influence of atmospheric turbulence and ground effect on the UAV. The authors of [39] synthesized the $H_{\infty }$ controller for both pitch and roll angles for a real quadcopter, first performing a continuous-time system identification on experimental data to capture a nominal model of the system as well as a multiplicative uncertainty.

Despite the robustness guarantees of the $H_{\infty }$ controller, its performance relies on a fixed controller structure, which cannot adapt to unknown disturbances or aggressive maneuvers. In such scenarios, a quadcopter exhibits strong and unpredictable nonlinear behaviors, compromising the nominal performance of the vehicle. To cope with these limitations, adaptive control techniques offer a viable solution [40,41,42]. Among adaptive methodologies, Model Reference Adaptive Control (MRAC) has attracted considerable attention for its ability to compensate for parametric uncertainties. First introduced in the 1950s by Whitaker [43], MRAC has been successfully applied by several researchers to quadrotor platforms. In [44], the MRAC with a modified version of the MIT rule is used to control the altitude of a quadcopter. The authors of [45] used a PID to control the motor speed while a direct MRAC was used for the high-level control of attitude angles. The use of the MRAC paradigm in the attitude loop of a hexacopter was proposed in [46] to adapt the control coefficients in case of failure or when the payload is released.

However, the classical MRAC-based control approach presents some limitations, such as dependence on precise models and sensitivity to parametric variations, especially in fast-dynamics applications [47,48,49]. To overcome these difficulties, several authors have focused on the use of neural network (NN)-based controllers, allowing them to create more robust and flexible adaptive models capable of managing in real time the uncertainties and nonlinearities typical of the behavior of a quadcopter [47,50].

In recent years, safety reasons have pushed researchers to develop hybrid architectures, combining a baseline controller with an NN compensator, with the latter able to provide an additive correction signal to enhance robustness and adaptability in the presence of model uncertainties and external disturbances [51]. Indeed, although NN-based controllers outperform traditional methods under external disturbances or model uncertainties once trained, the training phase itself requires a stable baseline control strategy [52]. The authors of [53] proposed a control scheme to stabilize the attitude of a mobile manipulating UAV (MM-UAV), using a combination of gain scheduling and Lyapunov-based MRAC. In [54], the authors presented a control architecture based on PID and MRAC to compensate for any UAV failures, with the MRAC controller able to provide an additive signal to guarantee the global asymptotic stability of the damaged system. The work proposed in [55] introduced a hybrid adaptive control technique based on MRAC as a compensator for the attitude control of a quadrotor, whose performance is analyzed in the presence of disturbances.

To further improve the capabilities of the above-mentioned approaches, $H_{\infty }$ robustness can be combined with learning techniques to take advantage of both approaches. For instance, the authors of [56] use Lyapunov analysis to treat network reconstruction errors as disturbances within an $H_{\infty }$ framework, utilizing monitoring logic to prevent system divergence. Likewise, the approach in [57] employs high-order neural networks to make nominal controllers more robust without requiring predefined bounds, at the cost of increased control effort and local validity. Additionally, in [58], the authors minimize computational burden through the adaptation of a single parameter, though this introduces the risk of chattering due to discontinuous control. In aeronautics, a combination of $H_{\infty }$ and neural adaptive control has been used to solve different tasks. In [59], an auto-landing controller is designed using a high-order $H_{\infty }$ coupled with adaptive compensation, whereas a quadcopter controller is presented and validated through simulations in [60]. The use of ridge functions for rocket autopilot in [61] reduces the number of tuning parameters; however, it necessitates the online update of multiple gain matrices. The aim of this paper is to bridge the gap between theoretical design and experimental validation on resource-constrained systems, while continuing to guarantee robust stability and performance.

A preliminary study on this topic was published by the authors as a conference paper in [62].

Specifically, the main contributions of this work are as follows:

- A two-tier hybrid architecture combining a mixed-sensitivity H∞ baseline controller, which guarantees robust stability and noise attenuation, with an online adaptive NN loop that compensates for external disturbances and parametric variations in real time.
- By adopting Displaced Rectified Linear Unit (DReLU) activations within a Lyapunov-stable adaptation framework, the controller adapts online from scratch with zero offline pre-training, achieving a reduction in embedded processor load compared to standard Radial Basis Function (RBF)-based networks.
- A simulation campaign maps the hyperparameter space (adaptation rate, σ-modification factor, and hidden-layer dimension), defining the trade-off between convergence rate, tracking precision, and avoidance of high-frequency oscillations.
- Real-time deployment and benchmark testing on the STM32F405 microcontroller of a Crazyflie 2.1+ micro-UAV (Bitcraze AB, Malmö, Sweden) subjected to persistent unbalancing torques induced by a $2\cdot 10^{-3}$ kg weight added on one arm of the drone at a distance of ≈$5\cdot 10^{-2}$ m. The drone is constrained to attitude motion by being mounted on a dedicated 3-degree-of-freedom tilting test rig with a spherical joint, resulting in negligible friction and inertia variation.

The paper is organized as follows: Section 2 introduces the UAV dynamics model while Section 3 describes the proposed flight control algorithm. Section 4 presents the results of the sensitivity analysis, carried out by changing the NN parameters, alongside experimental tests performed on the Crazyflie 2.1+ platform, to prove the effectiveness of the proposed control architecture. Finally, Section 5 draws the conclusions of the work.

## 2. Quadrotor Dynamic Model

**Figure 1:**
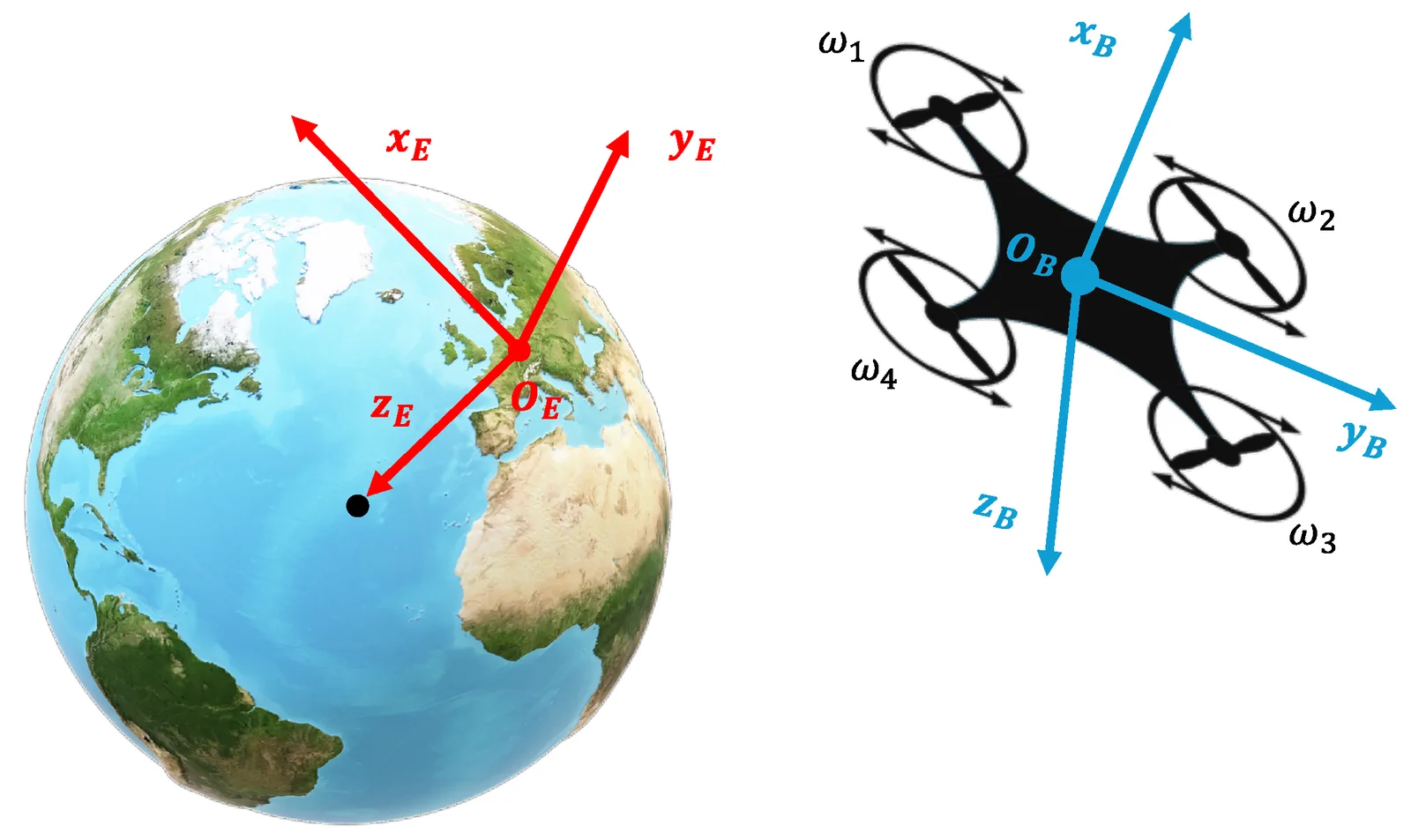
Coordinate control system definition: the Earth-fixed North–East–Down (NED) inertial frame $E=\{x_{E},y_{E},z_{E}\}$ and the aircraft body-fixed reference frame $B=\{x_{B},y_{B},z_{B}\}$ centered at the vehicle’s center of gravity.

The quadcopter dynamics are modeled by adopting Euler’s rigid body equations. Accordingly, the vehicle motion is described by considering its translational and rotational behavior with respect to two reference frames (see Figure 1):

- North–East–Down (NED) reference frame, $E=\{x_{E},y_{E},z_{E}\}$, located on the Earth’s surface;
- Body-fixed reference frame, $B=\{x_{B},y_{B},z_{B}\}$, whose origin, $O_{B}$, is located at the center of gravity (CG) of the quadrotor.

The transformation between these frames is expressed by the rotation matrix, $R_{BE}\left(\Theta \right)$ (Equation (1)), which maps the Earth-fixed frame to the body frame as a function of the UAV Euler angles $\Theta ={\left[\phi ,\theta ,\psi \right]}^{T}$,

(1) $$R_{BE}\left(\Theta \right)=\left[\begin{array}{ccc} C_{\theta }C_{\psi } & C_{\theta }S_{\psi } & -S_{\theta }\\ C_{\psi }S_{\theta }S_{\phi }-S_{\psi }C_{\phi } & S_{\psi }S_{\theta }S_{\phi }+C_{\psi }C_{\phi } & C_{\theta }S_{\phi }\\ C_{\psi }S_{\theta }C_{\phi }+S_{\psi }S_{\phi } & S_{\psi }S_{\theta }C_{\phi }-C_{\psi }S_{\phi } & C_{\theta }C_{\phi } \end{array}\right]$$

where $C_{\eta }=\cos\left(\eta \right)$ and $S_{\eta }=\sin\left(\eta \right)$, for η∈{ϕ,θ,ψ}.

Let $V={\left[u,v,w\right]}^{T}$ and $\Omega ={\left[p,q,r\right]}^{T}$ be the velocity and angular rate vectors expressed in the body frame, while F and T represent the external forces and torques acting on the vehicle with respect to the CG, respectively. The external forces depend on both the motion variables and the propellers’ rotational speeds, $\omega ={\left[\omega_{1},\omega_{2},\omega_{3},\omega_{4}\right]}^{T}$. Finally, let $p_{E}={\left[x_{E},y_{E},z_{E}\right]}^{T}$ represent the position of the quadrotor center of mass in the NED inertial reference frame.

The translational and rotational dynamics of the quadrotor are governed by Newton–Euler equations as follows:

(2) $$m\left(\dot{V}+\Omega \times V\right)=F\left(V,\Omega ,\Theta ,p_{E},\omega \right)$$

(3) $$J\dot{\Omega }+\Omega \times J\Omega =T\left(V,\Omega ,p_{E},\omega \right)$$

Equations (2) and (3) represent the translational and rotational equilibrium, respectively, where *m* is the mass of the quadrotor and J is the inertia matrix.

To complete the dynamic model, the relationships mapping the body-fixed velocities to the inertial frame derivatives are given by

(4) $$\dot{p_{E}}=R_{BE}^{-1}\left(\Theta \right)V$$

(5) $$\dot{\Theta }=H^{-1}\left(\Theta \right)\Omega$$

where the transformation matrix H(Θ) is defined as

(6) $$H\left(\Theta \right)=\left[\begin{array}{ccc} 1 & 0 & -\sin\theta \\ 0 & \cos\phi & \sin\phi \cos\theta \\ 0 & -\sin\phi & \cos\phi \cos\theta \end{array}\right]$$

The external forces and torques ((2) and (3)) can be decomposed into their physical contributions as follows:

(7) $$F\left(V,\Omega ,\Theta ,p_{E},\omega \right)=F_{g}\left(\Theta \right)+F_{p}\left(V,\Omega ,p_{E},\omega \right)+F_{a}\left(V,\Omega ,p_{E}\right)$$

(8) $$T\left(V,\Omega ,p_{E},\omega \right)=T_{p}\left(V,\Omega ,p_{E},\omega \right)+T_{a}\left(V,\Omega ,p_{E}\right)$$

where $F_{g}$ denotes the gravitational force, $F_{a}$ and $T_{a}$ account for aerodynamic forces and moments, respectively, expressed as nonlinear functions of the motion state [63]. The terms $F_{p}$ and $T_{p}$ denote the propulsive forces and moments generated by the combined action of the four motors, which constitute the primary control inputs of the vehicle.

**Figure 2:**
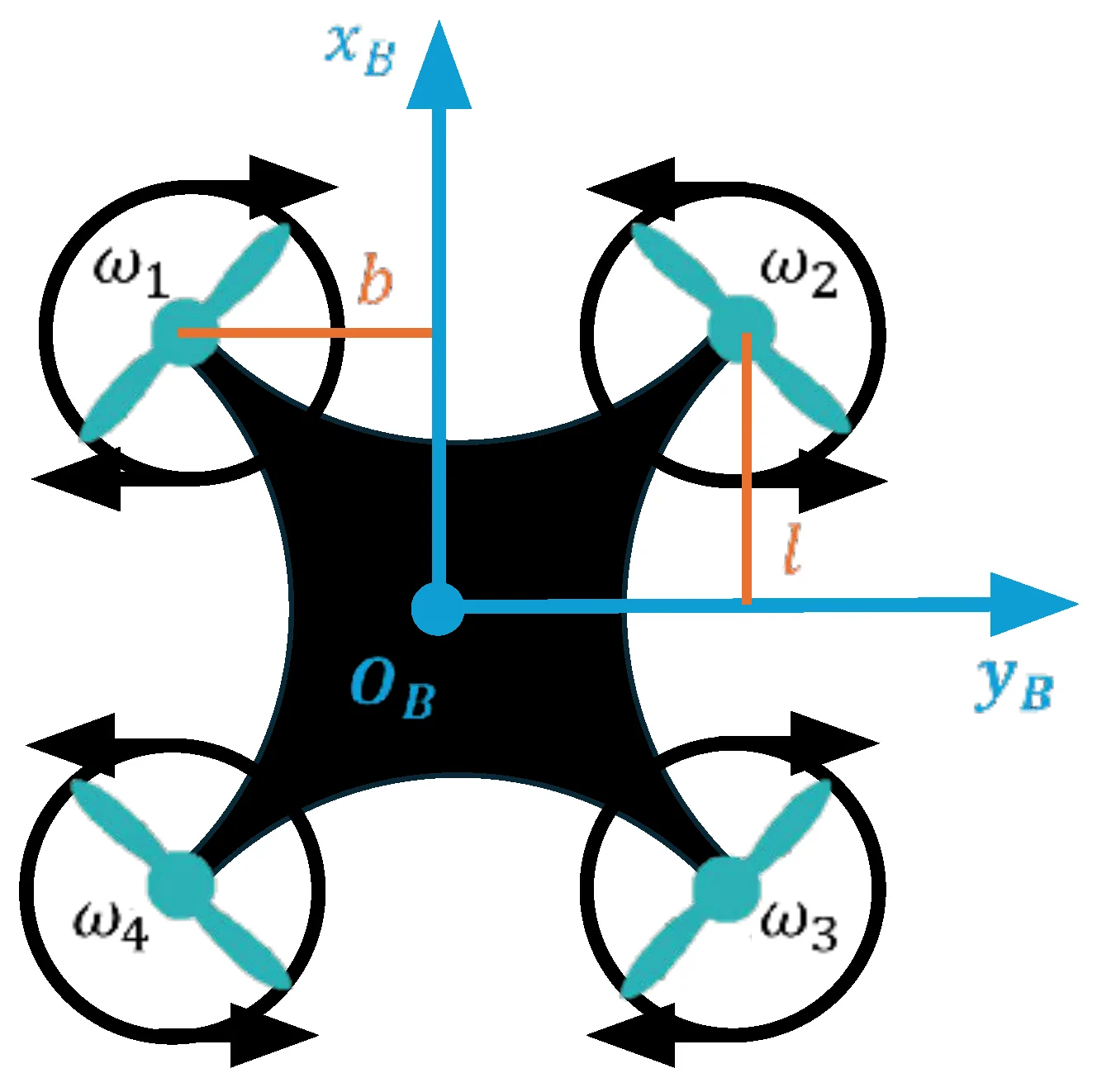
Schematic top view of the quadrotor airframe in X-configuration, detailing the counter-rotating propeller assignments ($\omega_{1}$ to $\omega_{4}$) and the respective structural moment arms (b,l) relative to the center of gravity.

Denoting with $F_{i}^{r}$ the thrust generated by the *i*-th rotor, the total propulsive forces and torques are expressed in the body-fixed frame as

(9) $$\left\{\begin{array}{c} F_{p}=-\sum_{i=1}^{4}F_{i}^{r}e_{3}\\ T_{p}=\left[\begin{array}{c} L_{p}\\ M_{p}\\ N_{p} \end{array}\right]=\left[\begin{array}{c} b[\left(F_{1}^{r}+F_{4}^{r}\right)-\left(F_{2}^{r}+F_{3}^{r}\right)]\\ l[\left(F_{1}^{r}+F_{2}^{r}\right)-\left(F_{3}^{r}+F_{4}^{r}\right)]\\ \sum_{i=1}^{4}\left[{\left(-1\right)}^{i+1}T_{i}^{r}\right] \end{array}\right] \end{array}\right.$$

where $e_{3}={\left[0,0,1\right]}^{T}$ is the unit vector along the *z*-axis of the body frame, $T_{i}^{r}$ represents the reactive torque produced by the *i*-th propeller, while *b* and *l* denote the distances of the rotor axes from the CG along the $y_{B}$ and $x_{B}$ axes, respectively (see Figure 2).

The forces and moments generated by the propellers depend on complex aerodynamic interactions and are generally difficult to model. However, near the hovering condition, a well-accepted simplified model expresses the thrust and drag torque of each rotor as a quadratic relation of its rotational speed $\omega_{i}$ via the coefficients $k_{f}$ and $k_{t}$ [64]. Under this assumption, the propulsive forces and moments can be compactly written in matrix form.

(10) $$\left[\begin{array}{c} F_{pz}\\ L_{p}\\ M_{p}\\ N_{p} \end{array}\right]=\left[\begin{array}{cccc} -k_{f} & -k_{f} & -k_{f} & -k_{f}\\ bk_{f} & -bk_{f} & -bk_{f} & bk_{f}\\ lk_{f} & lk_{f} & -lk_{f} & -lk_{f}\\ k_{t} & -k_{t} & k_{t} & -k_{t} \end{array}\right]\left[\begin{array}{c} \omega_{1}^{2}\\ \omega_{2}^{2}\\ \omega_{3}^{2}\\ \omega_{4}^{2} \end{array}\right]$$

The nonlinear quadrotor dynamics, defined in (2)–(5), can be linearized around a nominal steady-state hovering equilibrium. This operating condition is characterized by null translational and angular velocity vectors ($V_{0}=0$, $\Omega_{0}=0$), a constant nominal altitude $z_{E,0}$, and flat attitude profiles ($\Theta_{0}=0$), implying that the nominal propulsive thrust vector perfectly balances gravity.

To yield a tractable linear model for control synthesis, the following standard assumptions are introduced:

- The quadrotor structure is rigid and symmetric, implying a diagonal inertia matrix, i.e., $J=\mathrm{diag}(J_{xx},J_{yy},J_{zz})$;
- The CG coincides with the geometric center of the vehicle;
- Aerodynamic forces and moments, other than the primary propulsive inputs, are neglected due to the low-speed flight regime near the hover state.

Under these assumptions, the attitude perturbation variables remain small (ϕ≈0,θ≈0,ψ≈0), which reduces the kinematic transformation matrices introduced in (4) and (5) to identity operators, namely $R_{BE}\left(\Theta \right)\approx I_{3\times 3}$ and $H^{-1}\left(\Theta \right)\approx I_{3\times 3}$.

By defining the perturbation state vector as $x\left(t\right)={\left[\delta V^{T},\delta \Omega^{T},\delta {p_{E}}^{T},\delta \Theta^{T}\right]}^{T}\in R^{12}$ and the control input variations as $u\left(t\right)={\left[\delta F_{pz},\delta L_{p},\delta M_{p},\delta N_{p}\right]}^{T}\in R^{4}$, the resulting small-disturbance dynamics can be compactly formulated in the standard state–space representation:

(11) $$\begin{array}{c} \begin{array}{cc} \dot{x}\left(t\right) & =Ax(t)+Bu(t)\\ y(t) & =Cx(t)+Du(t), \end{array} \end{array}$$

where the state matrix $A\in R^{12\times 12}$ and the input matrix $B\in R^{12\times 4}$ are analytically derived via Jacobian evaluation at the hover state, yielding the following sparse block-partitioned structures:

(12) $$A=\left[\begin{array}{cccc} 0_{3\times 3} & 0_{3\times 3} & 0_{3\times 3} & A_{V\Theta }\\ 0_{3\times 3} & 0_{3\times 3} & 0_{3\times 3} & 0_{3\times 3}\\ I_{3\times 3} & 0_{3\times 3} & 0_{3\times 3} & 0_{3\times 3}\\ 0_{3\times 3} & I_{3\times 3} & 0_{3\times 3} & 0_{3\times 3} \end{array}\right],\;B=\left[\begin{array}{c} B_{V}\\ B_{\Omega }\\ 0_{3\times 4}\\ 0_{3\times 4} \end{array}\right]$$

where the non-zero sub-matrices capture the dominant local gravity coupling and control allocation channels, defined respectively as

(13) $$A_{V\Theta }=\left[\begin{array}{ccc} 0 & g & 0\\ -g & 0 & 0\\ 0 & 0 & 0 \end{array}\right],\;B_{V}=\left[\begin{array}{cccc} 0 & 0 & 0 & 0\\ 0 & 0 & 0 & 0\\ \frac{1}{m} & 0 & 0 & 0 \end{array}\right],\;B_{\Omega }=\left[\begin{array}{cccc} 0 & \frac{1}{J_{xx}} & 0 & 0\\ 0 & 0 & \frac{1}{J_{yy}} & 0\\ 0 & 0 & 0 & \frac{1}{J_{zz}} \end{array}\right]$$

Assuming full state availability, the output mapping matrices reduce to

(14) $$C=I_{12\times 12},\;D=0_{12\times 4}$$

## 3. Flight Control Algorithm

The scope of this paper is to analyze the attitude performance of a drone constrained to a platform. Consequently, a position controller is not required, and the remainder of the analysis focuses exclusively on the attitude dynamics.

To design the control algorithms for the attitude tracking loop, a reduced-order state space model is extracted from the linearized dynamics (11). Let $\tilde{x}\left(t\right)={\left[\delta \Theta^{T},\delta \Omega^{T}\right]}^{T}={\left[\delta \phi ,\delta \theta ,\delta \psi ,\delta p,\delta q,\delta r\right]}^{T}\in R^{6}$ denote the attitude state vector, and $\tilde{u}\left(t\right)={\left[\delta L_{p},\delta M_{p},\delta N_{p}\right]}^{T}\in R^{3}$ represent the virtual control moments. Incorporating additive sensor noise ν(t), the reduced-order attitude dynamics is formulated as

(15) $$\begin{array}{cc} \dot{\tilde{x}}\left(t\right) & =\tilde{A}\tilde{x}\left(t\right)+\tilde{B}\tilde{u}\left(t\right)\\ \tilde{y}\left(t\right) & =\tilde{C}\tilde{x}\left(t\right)+\nu \left(t\right) \end{array}$$

where the sparse system matrices $\tilde{A}\in R^{6\times 6}$, $\tilde{B}\in R^{6\times 3}$, and the measurement matrix $\tilde{C}\in R^{6\times 6}$ directly follow the structural partitions defined in (12).

**Figure 3:**
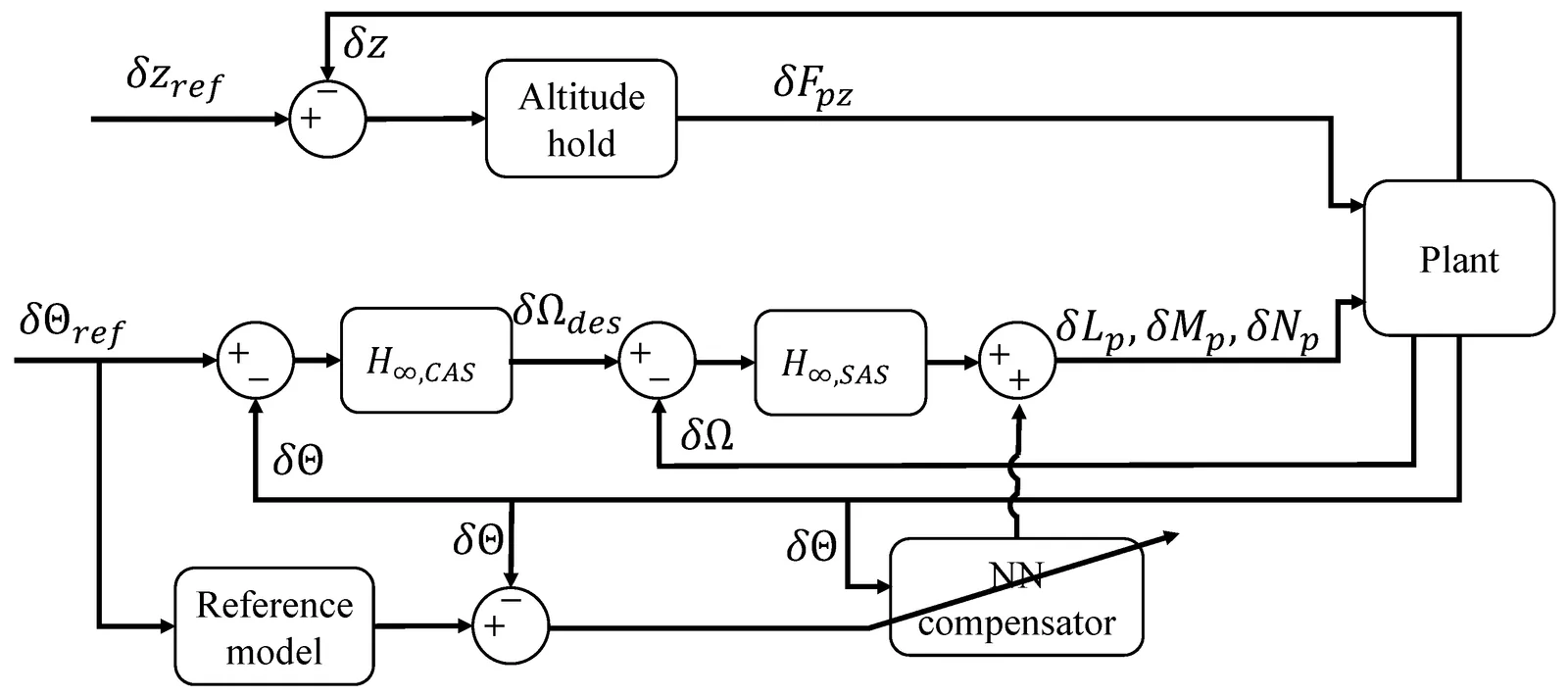
Global architecture of the proposed control system, illustrating the parallel decoupling layout between the inner attitude tracking loop augmented via the NN-based adaptive compensator and the baseline altitude-hold channel.

The proposed control architecture consists of a robust linear $H_{\infty }$ baseline controller augmented by an online adaptive neural network compensator. The general control architecture is shown in Figure 3. It has a cascade structure where the angular rate and the attitude feedback is separated to mimic the so-called Stability Augmentation System (SAS) and Control Augmentation System (CAS) [20].

To formalize this hierarchical structure, Equation (15) is partitioned into two vector equations corresponding to the attitude kinematics and the rotational dynamics:

(16) $$\begin{array}{cc} \delta \dot{\Theta }\left(t\right) & ={\tilde{A}}_{11}\delta \Theta \left(t\right)+{\tilde{A}}_{12}\delta \Omega \left(t\right)+{\tilde{B}}_{1}\tilde{u}\left(t\right)\\ \delta \dot{\Omega }\left(t\right) & ={\tilde{A}}_{21}\delta \Theta \left(t\right)+{\tilde{A}}_{22}\delta \Omega \left(t\right)+{\tilde{B}}_{2}\tilde{u}\left(t\right) \end{array}$$

where the state matrices are partitioned accordingly as $\tilde{A}=\left[\begin{array}{cc} {\tilde{A}}_{11} & {\tilde{A}}_{12}\\ {\tilde{A}}_{21} & {\tilde{A}}_{22} \end{array}\right]$ and $\tilde{B}=\left[\begin{array}{c} {\tilde{B}}_{1}\\ {\tilde{B}}_{2} \end{array}\right]$ with ${\tilde{A}}_{11},{\tilde{A}}_{21},{\tilde{A}}_{22},{\tilde{B}}_{1}=0$ and ${\tilde{A}}_{12}=I$.

The CAS (outer loop) acts on the attitude kinematics represented by the first row in (16). The output of this subsystem is a desired angular rate variation to drive the attitude tracking error to zero:

(17) $${\delta \Omega }_{des}\left(t\right)=\delta {\dot{\Theta }}_{ref}\left(t\right)+K_{CAS}e_{\Theta }\left(t\right)$$

where $e_{\Theta }=\delta \Theta_{ref}\left(t\right)-\delta \Theta \left(t\right)$ is the attitude tracking error and $K_{CAS}$ denotes the transfer function of the feedback controller $H_{\infty ,CAS}$.

$\delta \Omega_{des}$ is then fed to the SAS (inner loop, i.e., the second row of (16)) which computes the virtual control moments $\tilde{u}\left(t\right)={\left[\delta L_{P},\delta M_{P},\delta N_{P}\right]}^{T}$ that drive the angular rate error to zero:

(18) $${\tilde{u}}_{H_{\infty }}\left(t\right)={\tilde{B}}_{2}^{-1}\left(\delta {\dot{\Omega }}_{\mathrm{des}}\left(t\right)+K_{SAS}e_{\Omega }\left(t\right)\right)$$

where $e_{\Omega }=\delta \Omega_{des}\left(t\right)-\delta \Omega \left(t\right)$ is the tracking error of the angular rate, $K_{SAS}$ is the transfer function of the feedback controller $H_{\infty ,SAS}$ and $B_{2}=J^{-1}$.

**Figure 4:**
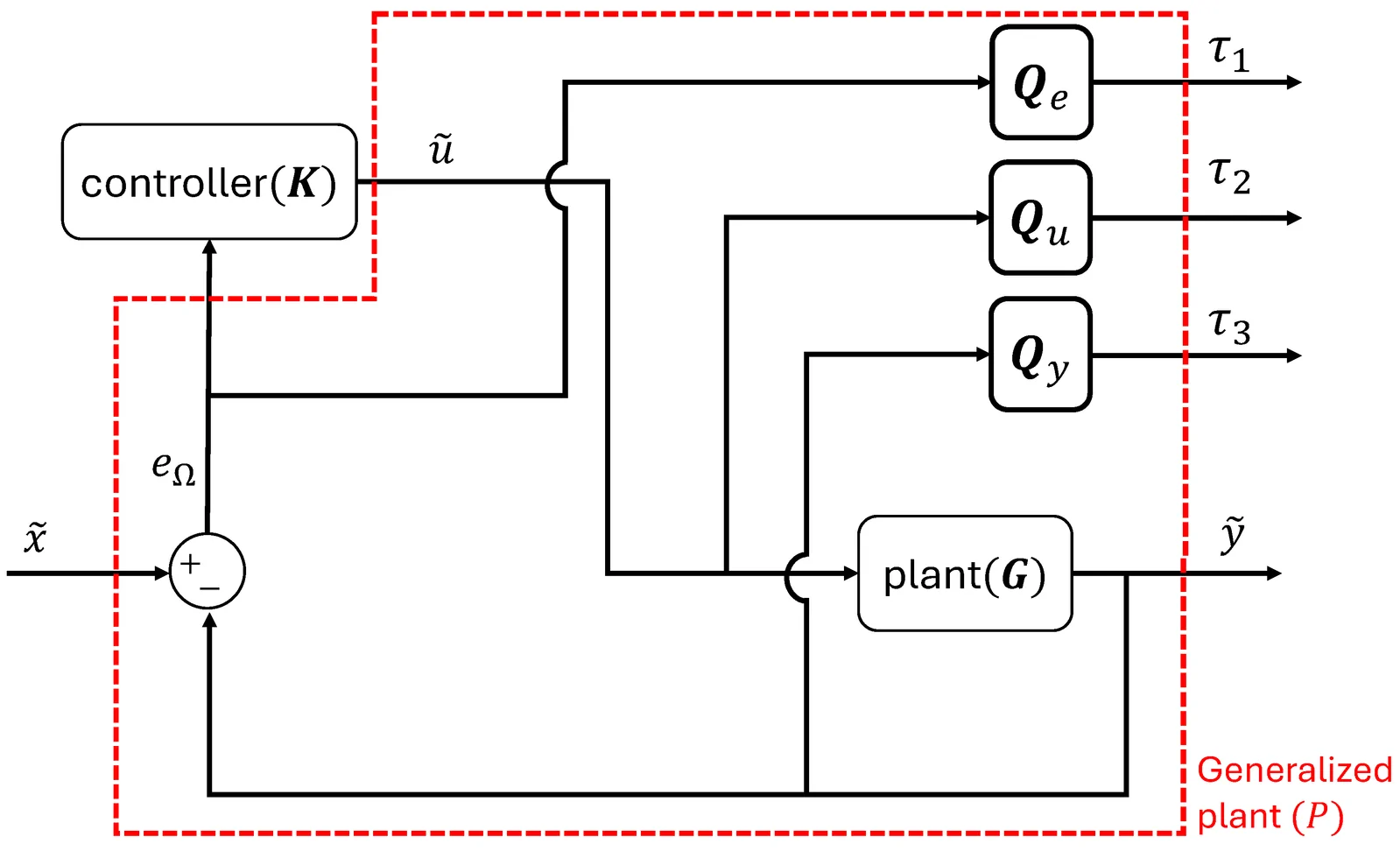
Standard mixed-sensitivity $H_{\infty }$ optimization framework setup, detailing the closed-loop interconnection of the reduced attitude plant G(s), the baseline controller K(s), and the dynamic shaping weight filters $Q_{e}\left(s\right)$, $Q_{u}\left(s\right)$, and $Q_{y}\left(s\right)$ embedded within the generalized plant P(s).

Each controller j∈{CAS,SAS} is synthesized within the mixed-sensitivity $H_{\infty }$ framework to satisfy tracking performance and robustness margins. Three dynamic weighting functions are considered to shape the closed-loop response (see Figure 4): $Q_{e,j}\left(s\right)$ to minimize steady-state tracking error, $Q_{u,j}\left(s\right)$ to penalize high-frequency control effort and $Q_{y,j}\left(s\right)$ to mitigate measurement noise injection. Each shaping filter is cast into a minimal state-space realization.

Consider

$$\begin{array}{c} {\tilde{x}}_{CAS}=\delta \Theta ,\;e_{CAS}=e_{\Theta },\;u_{CAS}=\delta \Omega_{des},\;y_{CAS}=\delta \Theta \\ {\tilde{x}}_{SAS}=\delta \Omega ,\;e_{SAS}=e_{\Omega },\;u_{SAS}={\tilde{u}}_{H_{\infty }},\;y_{SAS}=\delta \Omega \end{array}$$

By defining the augmented generalized state vector as $\xi_{j}={\left[{\tilde{x_{j}}}^{T},x_{e,j}^{T},x_{u,j}^{T},x_{y,j}^{T}\right]}^{T}$, the interconnection layout is encapsulated into the standard generalized plant P(s):

(19) $$\left[\begin{array}{c} \dot{\xi_{j}}\left(t\right)\\ \tau_{j}\left(t\right)\\ y_{K,j}\left(t\right) \end{array}\right]=\left[\begin{array}{ccc} A_{j} & B_{1,j} & B_{2,j}\\ C_{1,j} & D_{11,j} & D_{12,j}\\ C_{2,j} & D_{21,j} & D_{22,j} \end{array}\right]\left[\begin{array}{c} \xi_{j}\left(t\right)\\ R_{j}\left(t\right)\\ {\tilde{u}}_{j}\left(t\right) \end{array}\right]$$

where $\tau_{j}={\left[\tau_{1,j}^{T},\tau_{2,j}^{T},\tau_{3,j}^{T}\right]}^{T}$ represents the regulated performance outputs, $y_{K,j}$ is the input vector fed to the controller and $R_{j}$ is the reference signal. The dynamic output feedback control law is structured as $\tilde{u_{j}}\left(t\right)=K_{j}y_{K,j}\left(t\right)$.

The mixed-sensitivity synthesis [65] aims to compute an optimal stabilizing feedback controller $K_{j}$ that minimizes the $H_{\infty }$ norm of the closed-loop transfer matrix $Q_{R\tau ,j}:R_{j}\to \tau_{j}$:

(20) $$\min_{K_{j}}{∥Q_{R\tau ,j}\left(s\right)∥}_{H_{\infty }}\leq \gamma_{\infty ,j}$$

where $\gamma_{\infty ,j}>0$ denotes the disturbance attenuation performance bound and $Q_{R\tau ,j}\left(s\right)$ is structured as

(21) $$Q_{R\tau ,j}\left(s\right)=\left[\begin{array}{c} Q_{e,j}\left(s\right)S_{j}\left(s\right)\\ Q_{u,j}\left(s\right)K_{j}\left(s\right)S_{j}\left(s\right)\\ Q_{y,j}\left(s\right)T_{j}\left(s\right) \end{array}\right]$$

In Equation (21), $S_{j}\left(s\right)={\left(I+G_{j}\left(s\right)K_{j}\left(s\right)\right)}^{-1}$ and $T_{j}\left(s\right)=G_{j}\left(s\right)K_{j}\left(s\right){\left(I+G_{j}\left(s\right)K_{j}\left(s\right)\right)}^{-1}$ denote the sensitivity and complementary sensitivity functions of the nominal loop, respectively, with $G_{j}\left(s\right)$ representing the transfer matrix of the reduced attitude plant (15).

### Adaptive Neural Network Augmentation

To preserve tracking performance when operating far from the nominal hover condition or in the presence of severe unmodeled nonlinearities, the linear baseline control input ${\tilde{u}}_{H_{\infty }}\left(t\right)$ is augmented by an adaptive NN-based control scheme. Under unmodeled perturbations, the actual nonlinear attitude dynamics is formulated as

(22) $$\dot{\tilde{x}}\left(t\right)=\tilde{A}\tilde{x}\left(t\right)+\tilde{B}\Lambda \left({\tilde{u}}_{\Theta }\left(t\right)+f_{T}\left(\tilde{x}\right)\right)$$

where $\Lambda \in R^{3\times 3}$ is a positive-definite diagonal matrix modeling actuator effectiveness uncertainties, ${\tilde{u}}_{\Theta }\left(t\right)={\tilde{u}}_{H_{\infty }}\left(t\right)+u_{\mathrm{NN}}\left(t\right)$ is the total control command, and $f_{T}\left(\tilde{x}\right)\in R^{3}$ represents the lumped unmodeled nonlinearities and exogenous disturbances.

The control objective is to force the perturbed state $\tilde{x}\left(t\right)$ to asymptotically track the state $x_{r}\left(t\right)\in R^{6}$ of a stable linear reference model, defined by

(23) $${\dot{x}}_{r}\left(t\right)=A_{r}x_{r}\left(t\right)+B_{r}r\left(t\right)$$

where $A_{r}\in R^{6\times 6}$ is Hurwitz, $B_{r}\in R^{6\times 3}$, and $r\left(t\right)\in R^{3}$ is the bounded command input vector.

**Figure 5:**
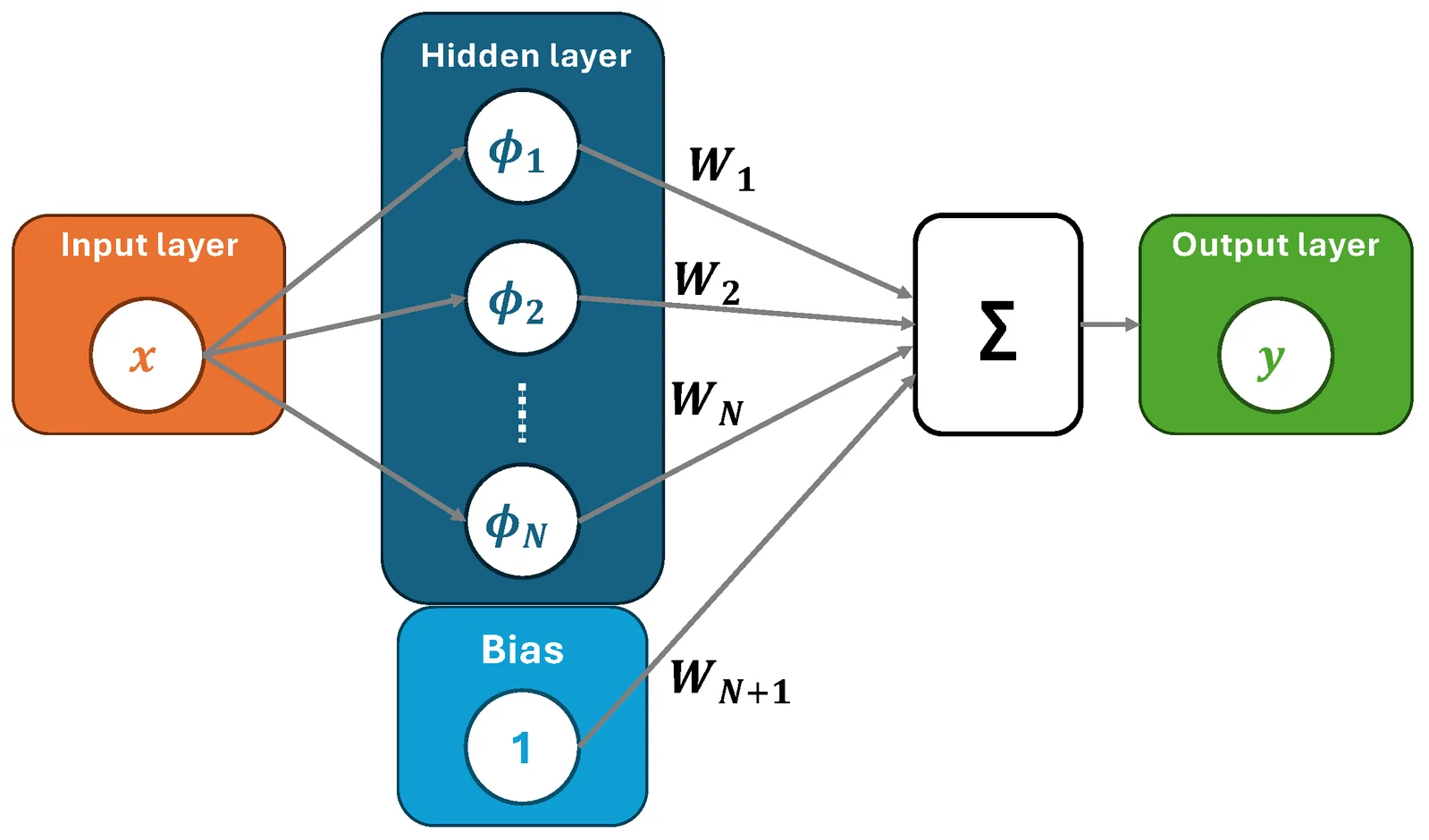
Structural topology of the augmented Single-Layer Feedforward NN compensator, showing the input layer, the hidden-layer neuron allocation ($\Phi_{1}$ to $\Phi_{N}$), the unit bias injection and the output-layer adaptive weight distribution W(t).

The adaptive compensation term $u_{\mathrm{NN}}\left(t\right)$ is parameterized via a Single-Layer Feedforward NN consisting of *N* hidden neurons and a linear output layer (see Figure 5):

(24) $$u_{\mathrm{NN}}\left(t\right)=-W^{T}\left(t\right)\Phi \left(\tilde{x}\right)$$

where $\Phi \left(\tilde{x}\right)={\left[\Phi_{1}\left(\tilde{x}\right),\ldots ,\Phi_{N}\left(\tilde{x}\right),1\right]}^{T}\in R^{N+1}$ defines the vector of activation functions augmented with a unit bias, and $W\left(t\right)\in R^{(N+1)\times 3}$ is the dynamically updated weight matrix:

(25) $$W\left(t\right)=\left[\begin{array}{ccc} w_{1,1}\left(t\right) & w_{1,2}\left(t\right) & w_{1,3}\left(t\right)\\ \vdots & \vdots & \vdots \\ w_{N+1,1}\left(t\right) & w_{N+1,2}\left(t\right) & w_{N+1,3}\left(t\right) \end{array}\right]$$

Let $e\left(t\right)=\tilde{x}\left(t\right)-x_{r}\left(t\right)$ and $e_{W}\left(t\right)=W_{opt}-W\left(t\right)$ denote the state tracking error and the weight estimation error, respectively. $W_{opt}$ represents the vector of optimal weight for which the ideal compensation term satisfies $W_{opt}^{T}\Phi \left(\tilde{x}\right)=f_{T}\left(\tilde{x}\right)$. The weight update law is designed by considering a Lyapunov function candidate V(t) that accounts for both tracking error and the weight estimation error. Due to the presence of neural network approximation error and external disturbances, the condition $\dot{V}\leq 0$ cannot be guaranteed everywhere. Instead, to add robustness and avoid the drift of neural network weights, a leakage term α is introduced, leading to a dissipative Lyapunov derivative inequality from which it is possible to demonstrate Uniform Ultimate Boundedness (UUB) of the tracking error [66,67,68].

(26) $$\dot{W}\left(t\right)=\Gamma_{\mathrm{NN}}\Phi \left(\tilde{x}\right)e^{T}\left(t\right)P\tilde{B}-\alpha W\left(t\right)$$

where $\Gamma_{\mathrm{NN}}>0$ represents the scalar adaptation rate, α>0 is the sigma-modification damping coefficient, and $P=P^{T}>0$ is the unique solution to the algebraic Lyapunov equation $A_{r}^{T}P+PA_{r}=-Q$, computed for a given symmetric positive-definite matrix Q.

## 4. Results

To assess the performance of the proposed architecture, a comprehensive evaluation campaign, comprising a numerical sensitivity analysis and experimental tests, has been carried out. The benchmarking platform chosen for this work is the Crazyflie 2.1+ micro-quadrotor, whose nominal geometric, inertial and mass parameters are summarized in Table 1.

Angles and angular rates are controlled in the baseline controller with a cascade structure. To design the control system, the weighting functions $Q_{e}\left(s\right)$, $Q_{u}\left(s\right)$, and $Q_{y}\left(s\right)$ are defined as first-order transfer functions.

The weighting matrices are structured in diagonal forms as

$$\begin{array}{ccc} Q_{e,CAS}\left(s\right) & = & \mathrm{diag}(\left[Q_{e,CAS}^{1},Q_{e,CAS}^{2},Q_{e,CAS}^{3}\right])\\ Q_{u,CAS}\left(s\right) & = & \mathrm{diag}(\left[Q_{u,CAS}^{1},Q_{u,CAS}^{2},Q_{u,CAS}^{3}\right])\\ Q_{y,CAS}\left(s\right) & = & \mathrm{diag}(\left[Q_{y,CAS}^{1},Q_{y,CAS}^{2},Q_{y,CAS}^{3}\right])\\ Q_{e,SAS}\left(s\right) & = & \mathrm{diag}(\left[Q_{e,SAS}^{1},Q_{e,SAS}^{2},Q_{e,SAS}^{3}\right])\\ Q_{u,SAS}\left(s\right) & = & \mathrm{diag}(\left[Q_{u,SAS}^{1},Q_{u,SAS}^{2},Q_{u,SAS}^{3}\right])\\ Q_{y,SAS}\left(s\right) & = & \mathrm{diag}(\left[Q_{y,SAS}^{1},Q_{y,SAS}^{2},Q_{y,SAS}^{3}\right]) \end{array}$$

The general structure used for $Q_{e,j}^{k}$ with k∈{1,2,3} is

(27) $$Q_{e,j}^{k}\left(s\right)=\epsilon_{j}^{k}\cdot \frac{s+\frac{M_{j}^{k}}{\epsilon_{j}^{k}}\cdot \omega_{j}^{k,b}}{s+\omega_{j}^{k,b}}$$

where $M_{j}^{k}$ denotes the low-frequency gain, $\epsilon_{j}^{k}$ represents the high-frequency gain, and $\omega_{j}^{k,b}$ is the target bandwidth frequency. The control input weights $Q_{u,j}^{k}\left(s\right)$ and the robustness weights $Q_{y,j}^{k}\left(s\right)$ share another generalized first-order formulation:

(28) $$Q_{u,j}^{k}\left(s\right)=G_{u,j}^{k,\infty }\cdot \frac{s+\omega_{u,j}^{k,z}}{s+\omega_{u,j}^{k,p}}$$

(29) $$Q_{y,j}^{k}\left(s\right)=G_{y,j}^{k,\infty }\cdot \frac{s+\omega_{y,j}^{k,p}}{s+\omega_{y,j}^{k,p}}$$

where $G_{u,j}^{k,\infty }$ is the asymptotic high-frequency gain. The zero frequency $\omega_{u,j}^{k,z}$ ($\omega_{y,j}^{k,z}$) and pole frequency $\omega_{u,j}^{k,p}$ ($\omega_{y,j}^{k,p}$) are uniquely specified by defining a target intermediate gain $G_{u,j}^{k,1}$ ($G_{y,j}^{k,1}$) at a transition frequency $\omega_{u,j}^{k,c}$ ($\omega_{y,j}^{k,c}$), alongside the low-frequency gain limit $G_{u,j}^{k,0}$ ($G_{y,j}^{k,0}$):

(30) $$\omega_{u,j}^{k,p}=\omega_{u,j}^{k,c}\sqrt{\frac{{\left(G_{u,j}^{k,1}\right)}^{2}-{\left(G_{u,j}^{k,0}\right)}^{2}}{{\left(G_{u,j}^{k,\infty }\right)}^{2}-{\left(G_{u,j}^{k,1}\right)}^{2}}},\;\omega_{u,j}^{k,z}=\omega_{u,j}^{k,p}\cdot \frac{G_{u,j}^{k,0}}{G_{u,j}^{k,\infty }}$$

(31) $$\omega_{y,j}^{k,p}=\omega_{y,j}^{k,c}\sqrt{\frac{{\left(G_{y,j}^{k,1}\right)}^{2}-{\left(G_{y,j}^{k,0}\right)}^{2}}{{\left(G_{y,j}^{k,\infty }\right)}^{2}-{\left(G_{y,j}^{k,1}\right)}^{2}}},\;\omega_{y,j}^{k,z}=\omega_{y,j}^{k,p}\cdot \frac{G_{y,j}^{k,0}}{G_{y,j}^{k,\infty }}$$

The complete set of parameters for both the inner (*p*,*q*,*r*-axes) and the outer loops (ϕ,θ,ψ-axes) is summarized in Table 2, Table 3 and Table 4. The $H_{\infty }$ bounds obtained with these parameters are $\gamma_{\infty ,CAS}\approx 167$ and $\gamma_{\infty ,SAS}\approx 2$.

To evaluate the tracking performance and statistical dispersion of the flight control system, the continuous-time states are discretized considering a constant sampling period $T_{s}$, such that the generic time instant is expressed as $t_{k}=kT_{s}$, where k∈N denotes the discrete-time sample index.

The reference model used to train the neural network is represented by the following transfer function:

(32) $$G_{ref}\left(s\right)=\frac{16}{s^{2}+8s+16}$$

Denoting with $\eta \left(k\right)≜\eta \left(kT_{s}\right)$ and $\eta_{\mathrm{ref}}\left(k\right)≜\eta_{\mathrm{ref}}\left(kT_{s}\right)$ the generic component of the actual and reference attitude vectors sampled at the *k*-th step, respectively, the discrete-time tracking error is defined as

(33) $$e_{\eta }\left(k\right)=\eta \left(k\right)-\eta_{\mathrm{ref}}\left(k\right)$$

To quantitatively assess the tracking accuracy over a finite observation time window composed of $n_{k}$ total samples, the Mean Absolute Error (MAE) and the Standard Deviation (SD) are introduced as performance metrics [69]:

(34) $$MAE_{\eta }=\frac{1}{n_{k}}\sum_{k=1}^{n_{k}}\left|e_{\eta }\left(k\right)\right|$$

(35) $$SD_{\eta }=\sqrt{\frac{1}{\left(n_{k}-1\right)}\sum_{k=1}^{n_{k}}{\left(e_{\eta }\left(k\right)-\mu_{\eta }\right)}^{2}}$$

where $\mu_{\eta }=\frac{1}{n_{k}}\sum_{k=1}^{n_{k}}e_{\eta }\left(k\right)$ represents the mean algebraic tracking error.

Moreover, the smoothness of the control signal is assessed through the Total Variation $\Delta_{TV}$ [70], defined as the sum:

(36) $$\Delta_{TV}=\sum_{k=1}^{n_{k}}\left|u_{k+1}-u_{k}\right|$$

where $u_{k}$ is the control signal at the *k*-th time step. A lower value of $\Delta_{TV}$ indicates a smoother control profile, which is desirable to minimize actuator chattering.

### 4.1. Numerical Sensitivity Analysis

The baseline robust $H_{\infty }$ synthesis loop was derived via a digital twin of the micro-UAV implemented within a MATLAB/Simulink R2025b (MathWorks, Natick, MA, USA) environment, operating at a fixed sample time of $T_{s}=2\times 10^{-3}\;s$. To model parametric uncertainties and exogenous disturbances, an unbalancing CG displacement was injected, generating an unmodeled persistent pitching and rolling moment of $M_{d}=9.81\cdot 10^{-4}\;N\cdot m$, which replicates a weight of $2\cdot 10^{-3}$ kg mounted at $5\cdot 10^{-2}$ m from the CG.

To ensure enhanced distribution capability within the disturbance domain, the adaptive neural network augmentation utilizes DReLU, a piecewise linear activation function, which is an enhanced version of the standard Rectified Linear Unit (ReLU) function [71].

To address cross-channel coupling terms, a multidimensional neural network mapping the six-dimensional state $\tilde{x}$ is needed. However, in this paper, to reduce RAM consumption, cross-channel couplings are considered negligible and three separate NNs are used, one for every angle, mapping the experienced disturbance to a specific attitude configuration. The activation function is parameterized as

(37) $$\mathrm{DReLU}\left(x\right)=\max\left(\zeta_{\mathrm{bias}},x\right)$$

where $\zeta_{\mathrm{bias}}\in \left[-\frac{\pi }{6}:\frac{\pi }{6}\right]$ represents the specific activation threshold.

The numerical validation campaign is structured to replicate the real-time online learning behavior of the adaptive controller, emphasizing its ability to operate without any prior offline pre-training phase. At the beginning of the simulation, the NN weights are initialized to zero (W(0)=0), meaning that the adaptive component provides no control action initially, leaving the stabilization completely to the baseline $H_{\infty }$ loop.

Since the network weights must be updated dynamically from scratch in-flight, the simulation profile consists of an uninterrupted flight sequence divided into two subsequent operational phases: an initial online training window and a subsequent closed-loop evaluation phase. While the neural network learning remains continuously active, the division of the flight profile into training and evaluation phases emulates a realistic operational scenario. Specifically, before the actual flight, the adaptive compensator is given 82s to learn the disturbances, after which a hover is maintained for an additional 18s to evaluate learning retention.

**Figure 6:**
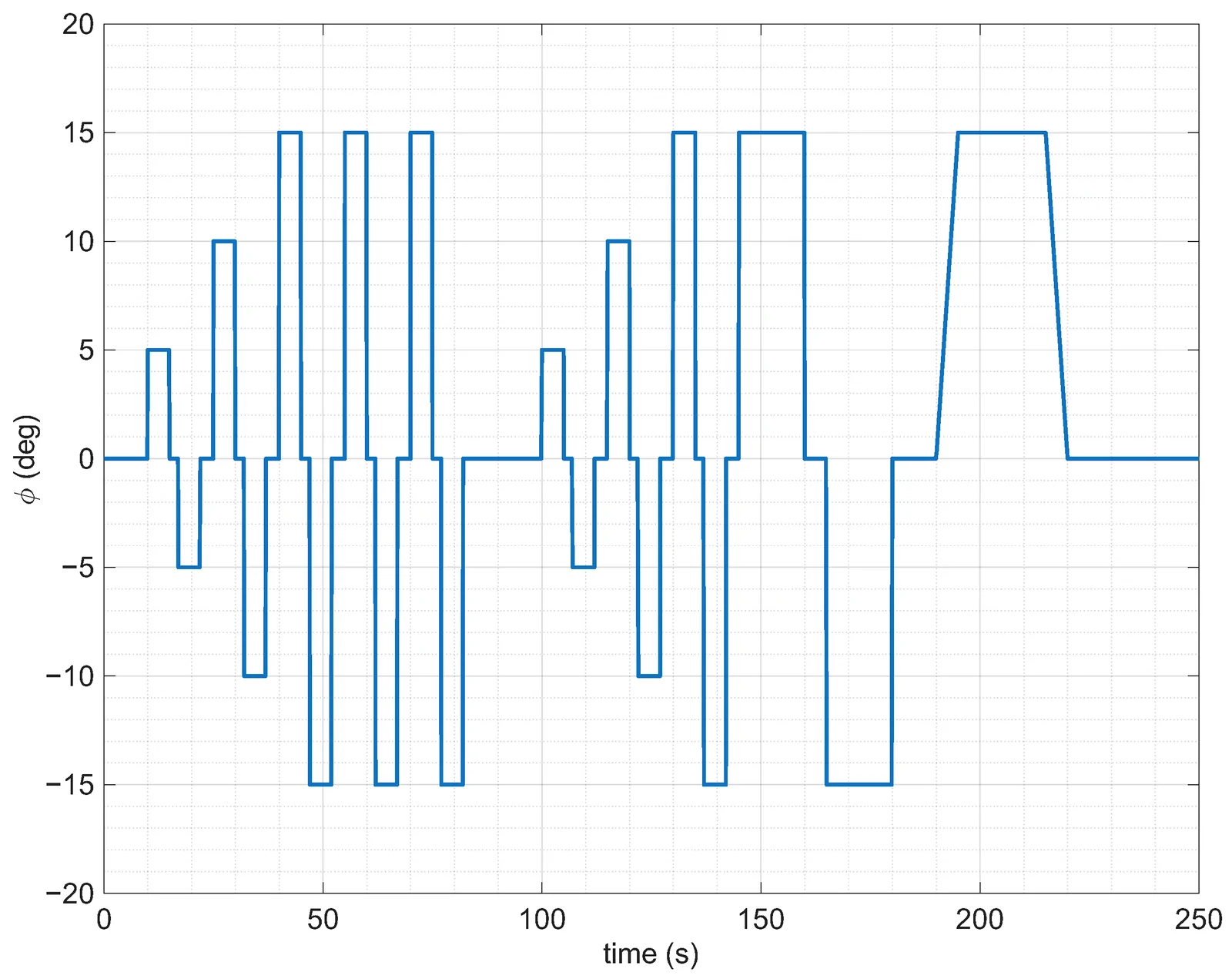
Multi-step reference command history $\phi_{\mathrm{ref}}\left(t\right)$ applied across the continuous time window, comprising the initial online adaptation doublets (0s≤t≤90s), the closed-loop evaluation doublets (90s≤t≤190s), and the final smoothness tracking trapezoidal profile.

Specifically, the training profile comprises five consecutive doublets with increasing peak-to-peak amplitudes of 10 deg, 20 deg, and 30 deg, with a period of $T_{d}=5\;s$. The evaluation test phase incorporates a cascade of four doublets (10 deg, 20 deg, 30 deg at $T_{d}=5\;s$ and a persistent 30 deg pulse of $T_{d}=15\;s$), followed by a trapezoidal command profile with a constant rate of 3 deg/s (see Figure 6), which is used to evaluate the performance metrics to avoid taking into account the jump of the doublets.

#### 4.1.1. Sensitivity Analysis #1: Hyperparameter Tuning ($\Gamma_{\mathrm{NN}}$, α)

A preliminary investigation was performed to characterize the joint influence of the learning rate $\Gamma_{\mathrm{NN}}$ and the sigma-modification decay parameter α on tracking capability. The analyzed grid configuration is specified in Table 5.

**Figure 7:**
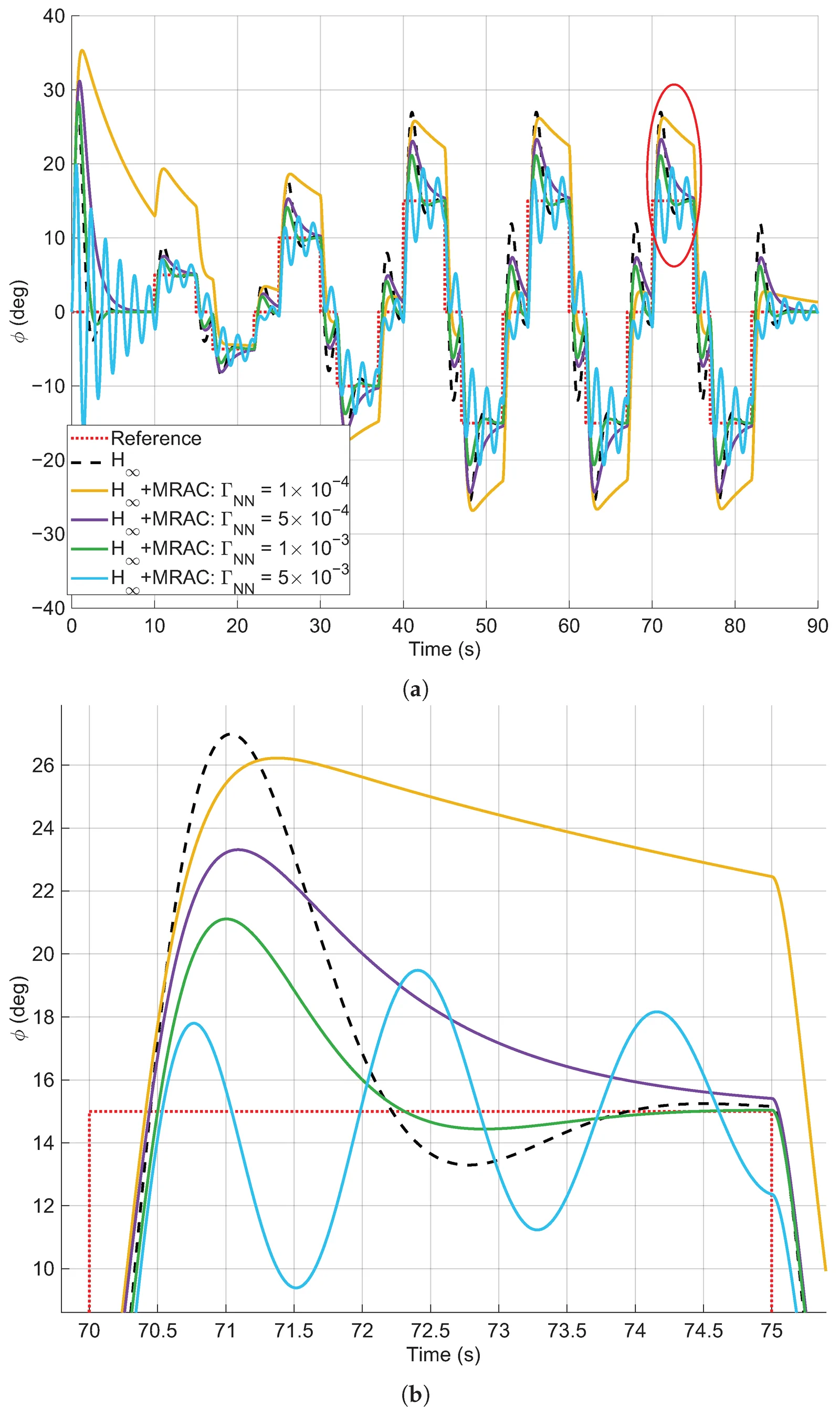
(**a**) Transient tracking response of the roll angle ϕ(t) during the online training phase across a range of adaptation rates $\Gamma_{\mathrm{NN}}$ (with $\alpha =1\times 10^{-4}$); the red circle indicates the region magnified in (**b**). (**b**) The magnified section highlights the settling behavior and high-frequency oscillations at peak values.

**Figure 8:**
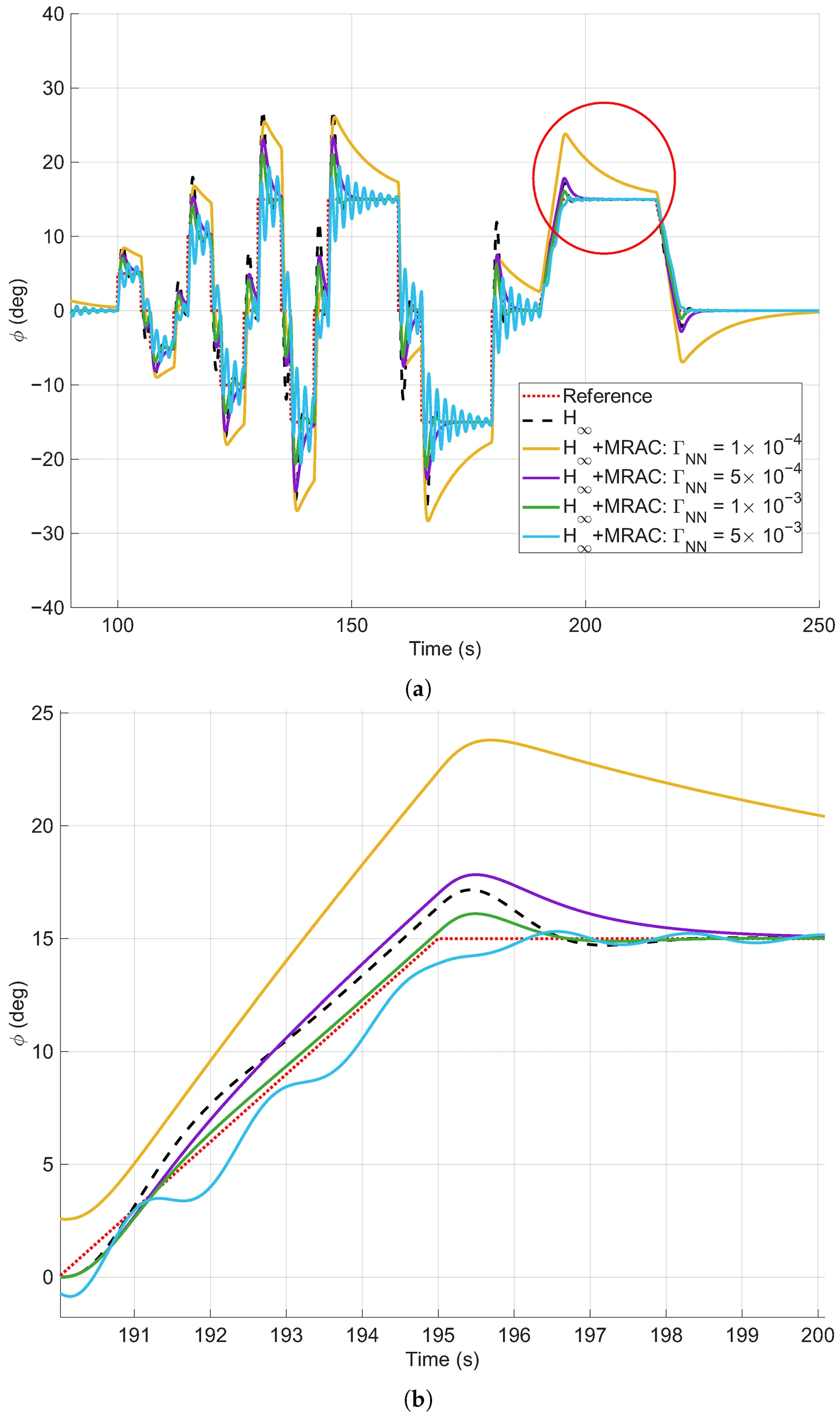
(**a**) Closed-loop attitude response validation of the roll angle ϕ(t) during the testing phase across different adaptation rates $\Gamma_{\mathrm{NN}}$ (with $\alpha =1\times 10^{-4}$); the red circle indicates the region magnified in (**b**). (**b**) The magnified section highlights tracking offsets and steady-state error mitigation on the persistent step command.

**Figure 9:**
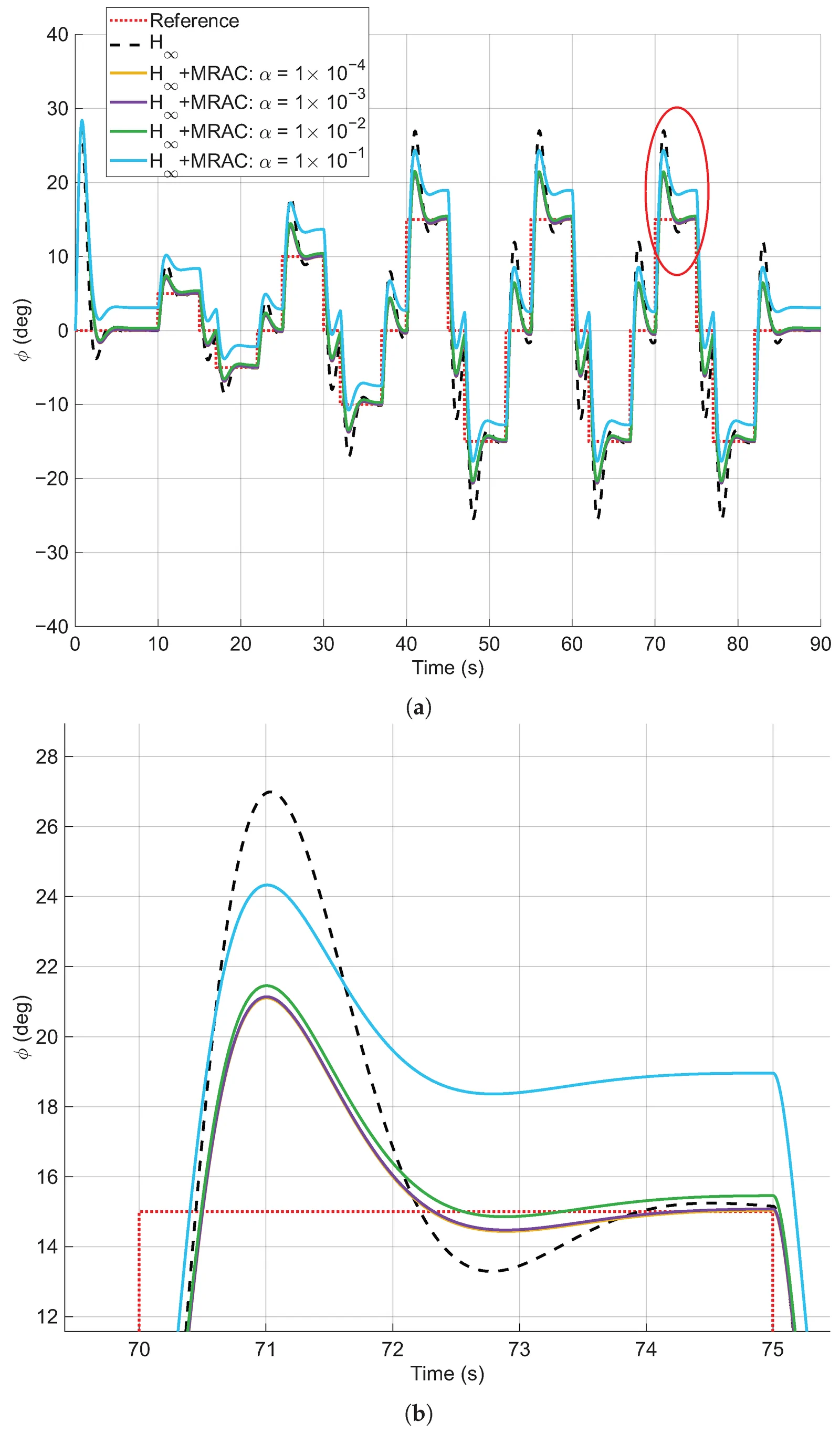
(**a**) Transient tracking response of the roll angle ϕ(t) during the online training phase across different leakage damping parameter α (with $\Gamma_{\mathrm{NN}}=1\times 10^{-3}$); the red circle indicates the region magnified in (**b**). (**b**) The magnified section emphasizes the drift suppression transient at t=70s.

**Figure 10:**
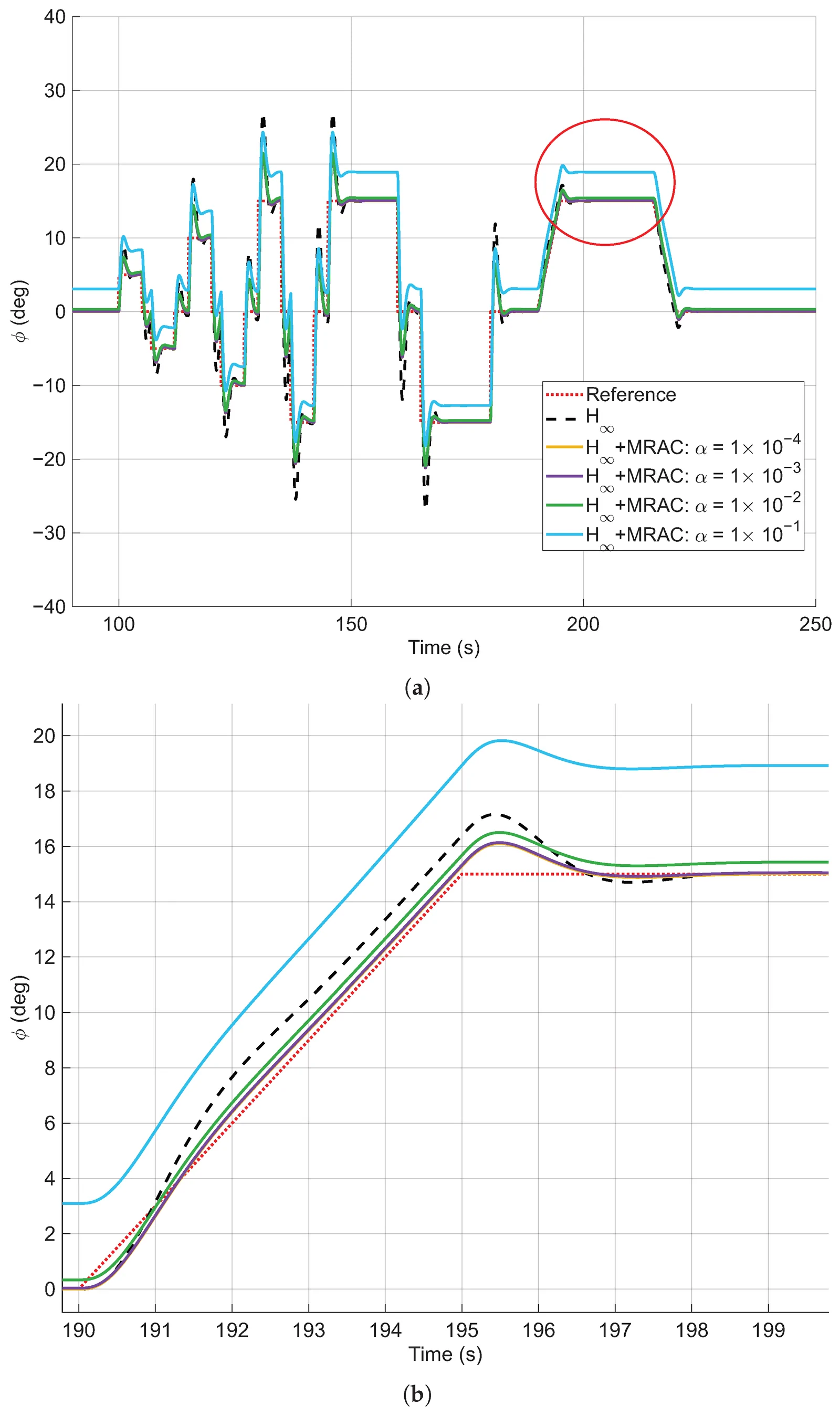
(**a**) Closed-loop attitude response validation of the roll angle ϕ(t) during the testing phase for different values of the leakage damping parameter α (with $\Gamma_{\mathrm{NN}}=1\times 10^{-3}$); the red circle indicates the region magnified in (**b**). (**b**) The magnified section details the degradation in tracking precision caused by excessive weighting.

The closed-loop tracking behavior during both the training and testing phase is illustrated in Figure 7, Figure 8, Figure 9 and Figure 10. As shown in Figure 7 and Figure 8, a lower adaptation gain ($\Gamma_{\mathrm{NN}}=1\times 10^{-4}$) slows down the error convergence rate, yielding worse tracking profiles. Conversely, excessive values ($\Gamma_{\mathrm{NN}}=5\times 10^{-3}$) induce high-frequency oscillatory components and chattering phenomena as shown by the increment in $\Delta_{TV}$, compromising the local loop stability. On the other hand, excessive leakage regulation via the decay factor α generates a more regular signal, as shown by the decrease in SD, at the cost of a steady-state tracking error resulting in a higher MAE.

These results are confirmed by the numerical indices shown in Table 6 and Table 7. Significant metric degradation occurs for hyperparameter choices exceeding $\Gamma_{\mathrm{NN}}>1\times 10^{-3}$ and $\alpha >1\times 10^{-3}$.

#### 4.1.2. Sensitivity Analysis #2: Hidden-Layer Sizing (*N*)

The second campaign focused on optimizing the hidden-network-layer dimension, exploring N∈[5,10,15,20,25,30,35].

**Figure 11:**
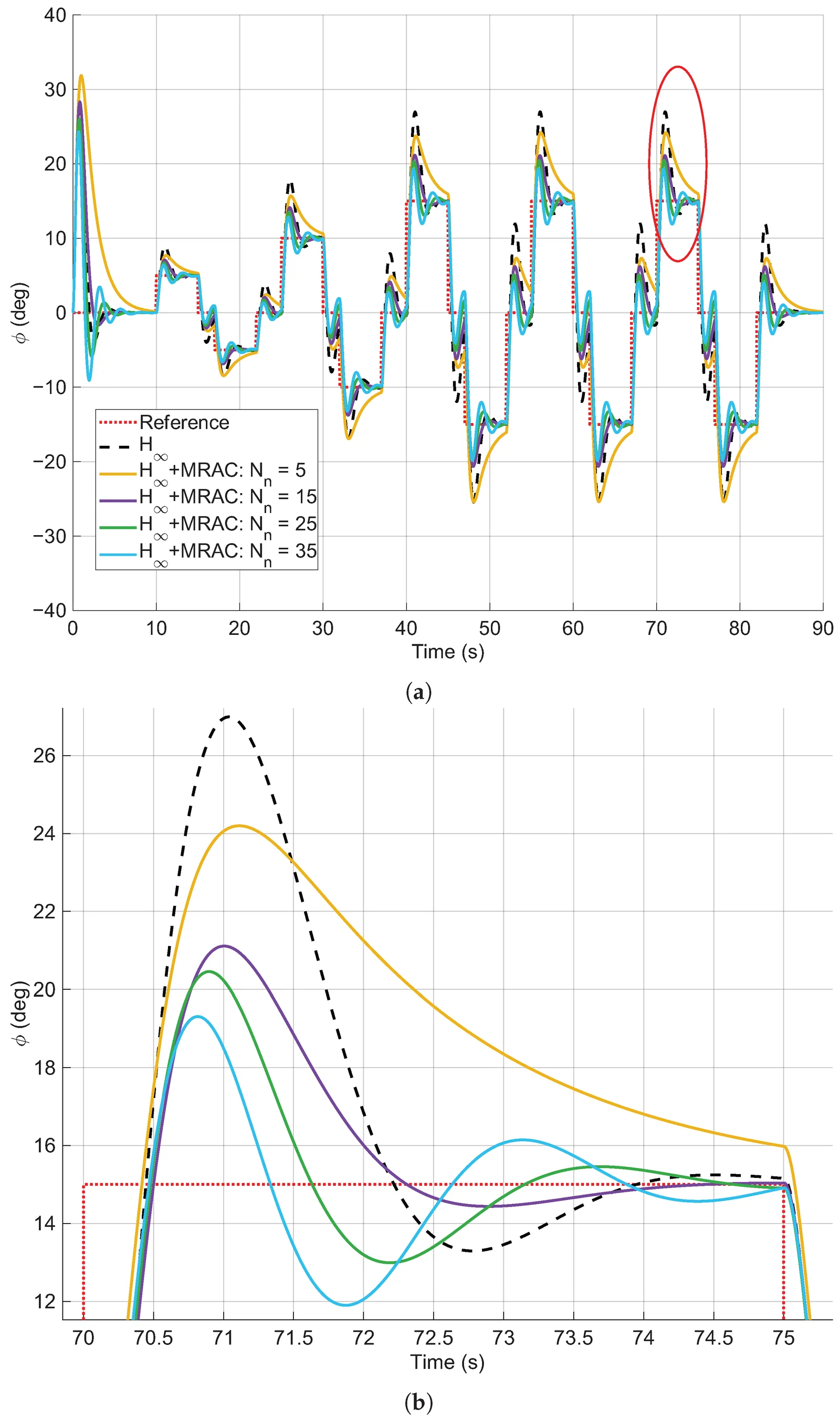
(**a**) Training phase response under variations in the hidden-layer size *N*; the red circle indicates the region magnified in (**b**). (**b**) The magnified plot emphasizes the under-modeling behavior of N=5 versus the optimal convergence of the selected N=15 node topology.

**Figure 12:**
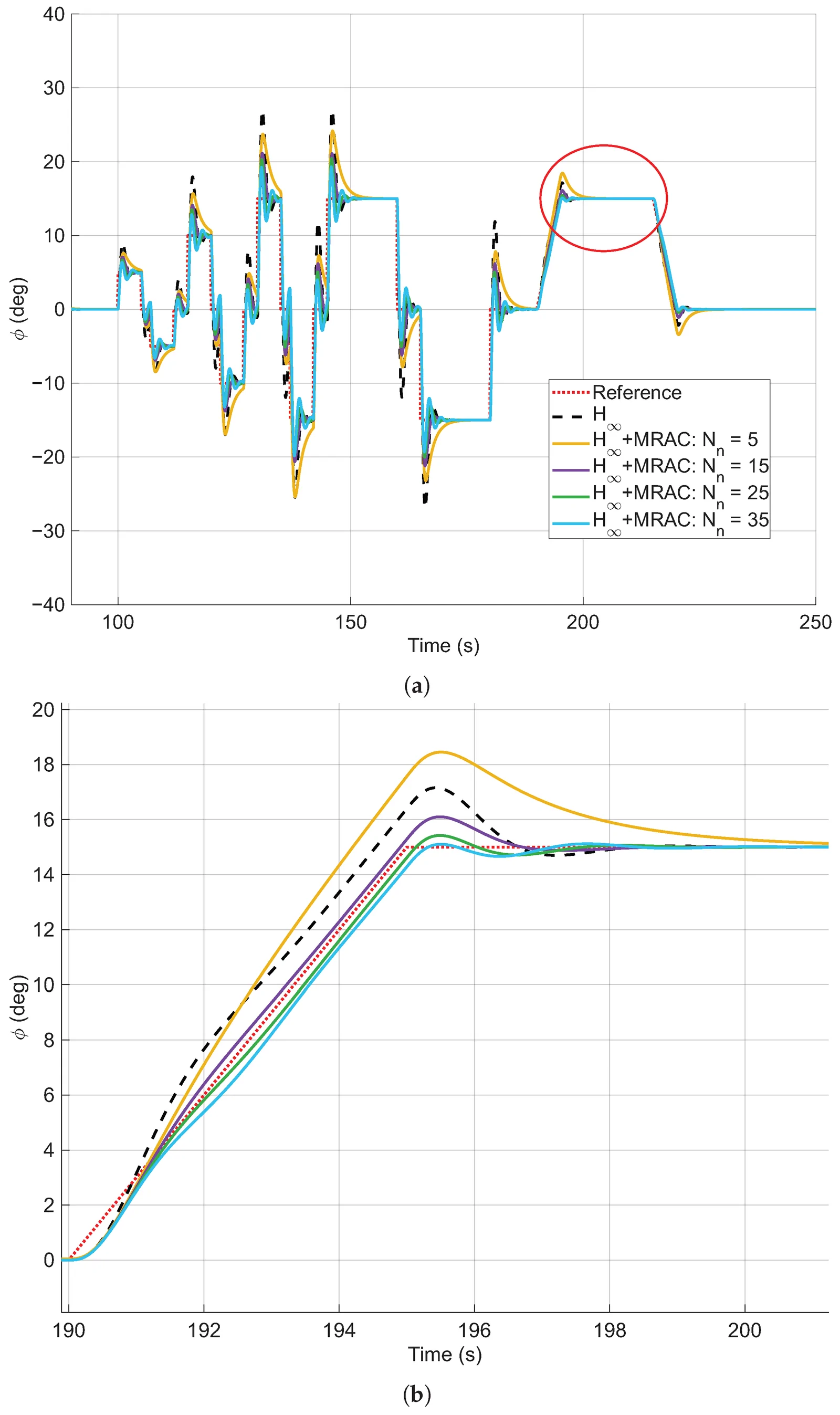
(**a**) Tracking accuracy validation during the evaluation phase across different hidden-layer configurations *N*; the red circle indicates the region magnified in (**b**). (**b**) The magnified plot highlights tracking degradation and oscillatory issues occurring at higher dimensions (N≥25).

The comparison is depicted in Figure 11 and Figure 12, while Table 8 reports the comparison in terms of the performance indices, defined in (34) and (35), considering different hidden-layer configurations.

Increasing the hidden dimension up to N=15 significantly enhances approximation capacity. However, expanding the dimension further (N≥25) triggers local high-frequency oscillations given the selected baseline parameters ($\Gamma_{\mathrm{NN}},\alpha$), leading to performance limitations [72].

As depicted in Table 8, while the steady-state tracking error saturates, the Total Variation $\Delta_{TV}$ further increases, indicating that the over-parameterized network attempts to fit high-frequency transient tracking errors and measurement noise rather than purely reconstructing the underlying lumped disturbance, resulting in actuator chattering. Since persistent excitation (PE) cannot be guaranteed uniformly across every discrete DReLU region during rapid transient maneuvers, an excessive number of localized basis functions allows individual weights to rapidly over-compensate for instantaneous transient errors along the trajectory. This leads to neural coupling transients before the σ-modification damping term is able to stabilize the weights.

While the configurations for 15 and 20 neurons yield close metric performance, the N=15 topology was selected for deployment. This choice is the result of practical design trade-off between performance and resource requirements: while increasing the number of neurons past 15 results in a reduction in MAE (as shown in Table 8), using fewer neurons translates into a smaller number of adaptive parameters to store and compute at each controller cycle, reducing memory usage and computational burden. Moreover, with less neurons, a better damped signal during transient response is achieved, as shown in Figure 11.

### 4.2. Experimental Results

To demonstrate the real-time feasibility of the proposed approach, the DReLU-parameterized network was implemented on the embedded architecture of a Crazyflie 2.1+ micro-UAV and benchmarked against a literature-standard RBF adaptive scheme [73]. The Crazyflie firmware was compiled using the arm-none-eabi-gcc toolchain targeting the ARM Cortex-M4 architecture with the size-optimization flag -Os (as specified in the standard firmware). RBF architectures provide localized function approximation fields written as

(38) $$\mathrm{RBF}\left(x\right)=e^{-\sigma {\left(x+\beta \right)}^{2}}$$

where σ and β represent the RBF kernel width and center vector, respectively. Despite their mathematical flexibility, RBF schemes scale poorly with hardware constraints due to high computational overhead of evaluating exponential functions. The baseline synthesis parameter sets for both architectures are organized in Table 9, featuring parameters adapted from standard verification configurations [74]. It is worth noting that the two neural networks have comparable architecture as they use the same number of weights and neurons, placed at identical positions to map the space equivalently.

A comparison between the baseline controller and the baseline-plus-compensation architecture was omitted because the standard $H_{\infty }$ controller employed here lacks an integral component, making such a benchmark unfair.

**Figure 13:**
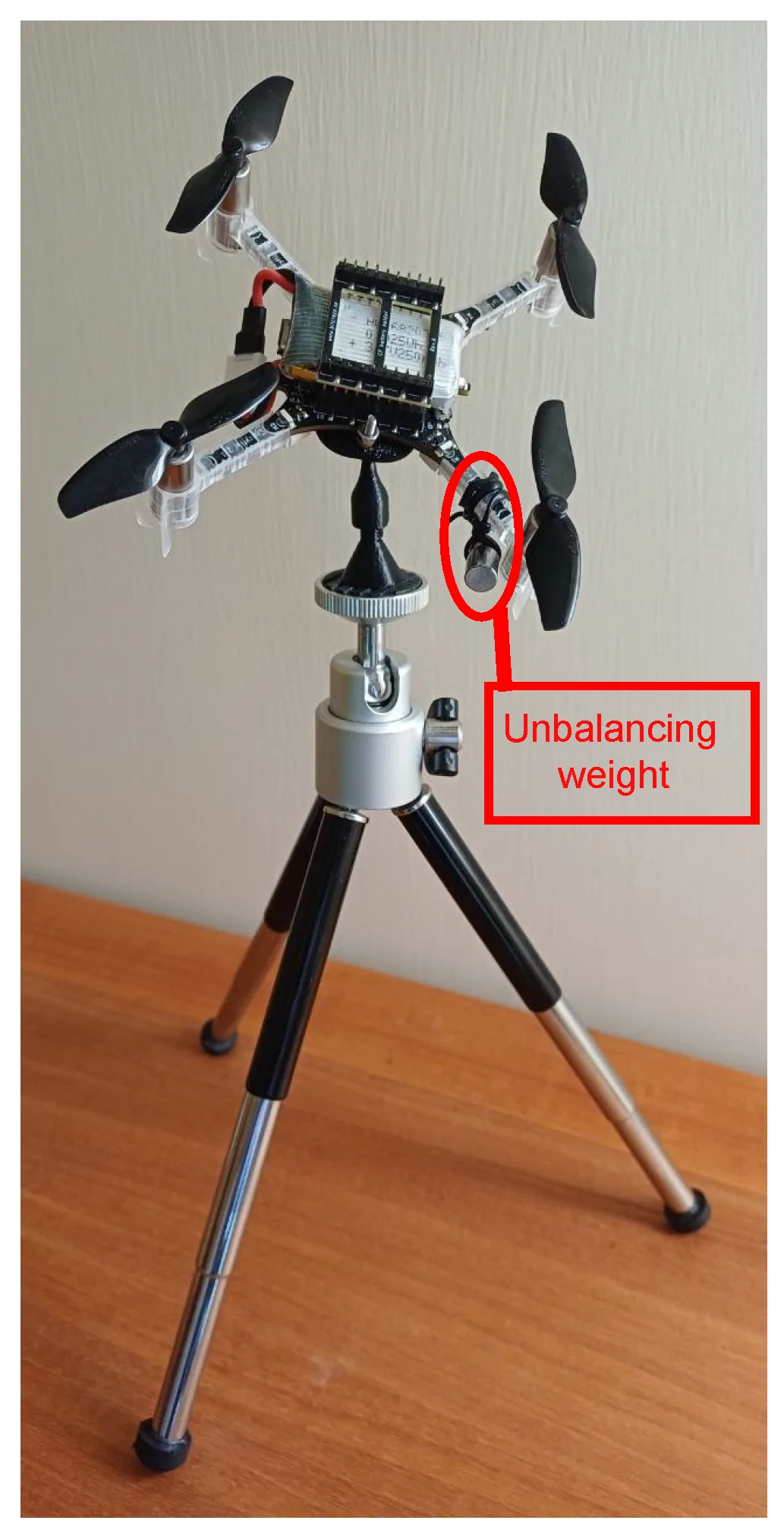
Real-time validation testbench setup: the Crazyflie 2.1+ micro-UAV is mounted to a low-friction tilting gimbal system restricting the vehicle to rotational attitude motions, featuring a $2\times 10^{-3}\;\mathrm{kg}$ calibrated offset payload to generate an asymmetrical unmodeled disturbance moment.

The test setup used a low-friction tilting calibration platform restricting the vehicle to a three-degrees-of-freedom constrained rotational body (Figure 13). The unmodeled asymmetrical disturbance torque was physically generated by adding a calibrated payload mass of $2\times 10^{-3}\;\mathrm{kg}$ beneath the M1 rotor axis at $\approx 5\cdot 10^{-2}$ m from the center of mass.

The training and test phases use the same reference signals as the numerical assessment depicted in Figure 6.

**Figure 14:**
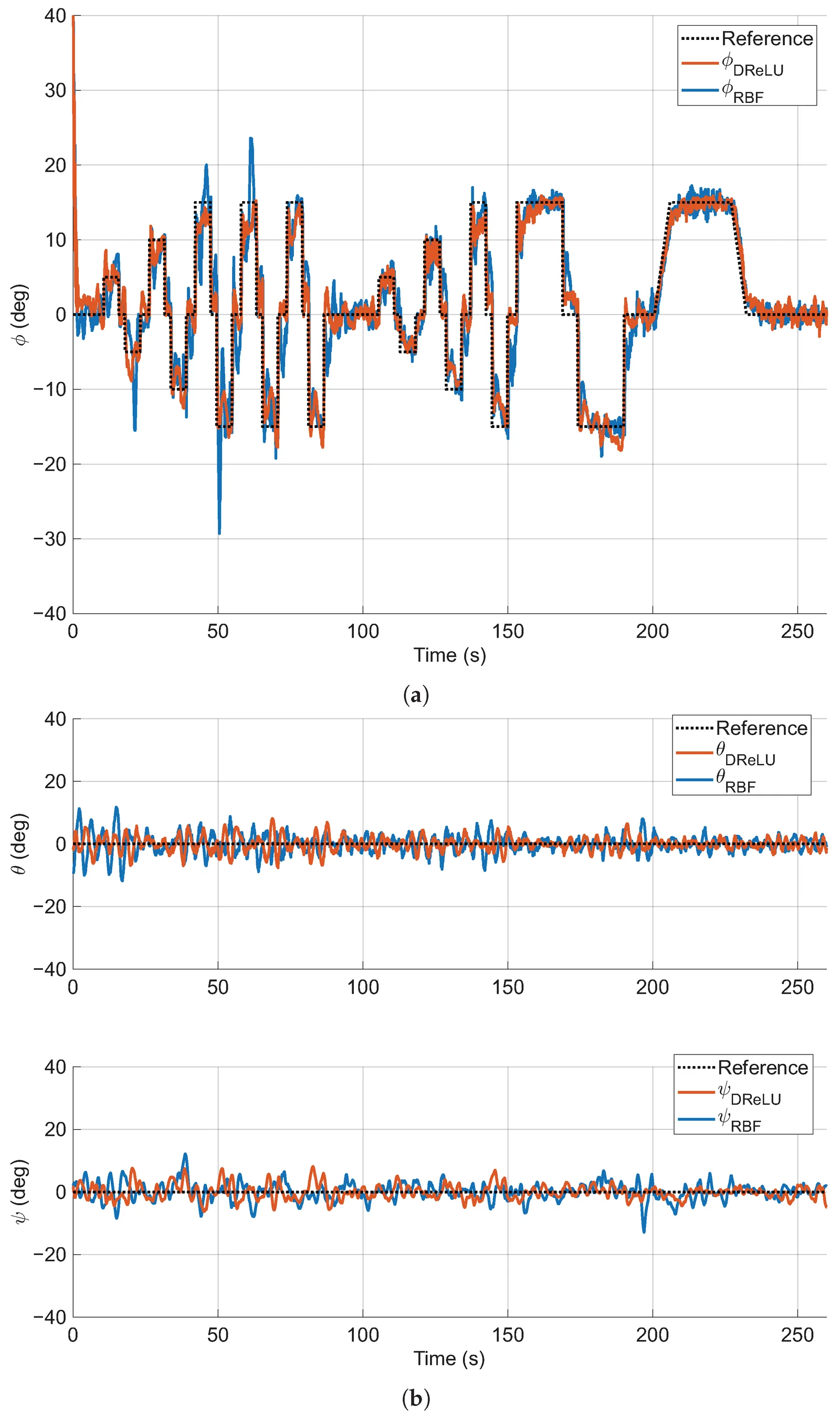
Experimental real-time flight telemetry histories (Roll ϕ (**a**) given the reference signal of Figure 6; Pitch θ and Yaw ψ (**b**) given a constant zero reference signal) under persistent unbalancing moment, contrasting the baseline $H_{\infty }$ controller performance augmented via the proposed linear DReLU network against the augmentation with the traditional RBF framework.

The closed-loop control histories for both validation schemes are shown in Figure 14. Both adaptive architectures successfully suppress the persistent unbalancing roll moment, showing minimal overshoots and negligible cross-channel coupling into the pitching and yawing fields.

In both cases, the memory usage is linked to the number of adaptive weights updated online, impacting the RAM. With 15 neurons, considering floating point representation, the neural network occupies only 60 bytes, which is a negligible percentage of the total memory of the considered processor. The primary distinction lies in the processing costs. Table 10 and Table 11 summarize the results, computed as the mean of ten independent tests. To measure the computational load of the two controllers running at a fixed frequency of 250Hz, the execution time of the entire control task was measured at each cycle using hardware timers and compared against the measured duration of the Central Processing Unit (CPU) cycle. The CPU utilization metrics—measured directly on the STM32F405 microcontroller (ARM Cortex-M4 operating core)—highlight that the linear nature of the DReLU network maintains the execution burden at 1.464%, achieving a 52.6% reduction in computational resource consumption relative to the RBF structure (3.094%) without sacrificing tracking quality.

## 5. Conclusions

This paper presents the design, implementation and experimental validation of an augmented robust flight control architecture tailored for resource-constrained micro-UAV platforms. In particular, this research focuses on attitude control by validating the algorithms on a custom platform, thus avoiding free flight tests and the need for a position controller. This work extends a preliminary study [62], where the use of a neural network compensator was discussed and a baseline PID was compared to a PID+NN architecture. The proposed framework integrates a baseline robust linear $H_{\infty }$ optimal stabilizer with an online NN-based loop parameterized via a Single-Layer Feedforward NN. By exploiting DReLU activation functions, the adaptive compensator ensures real-time mitigation of severe unmodeled nonlinearities and persistent exogenous perturbations while maintaining an extremely low computational footprint, making it fully compatible with low-power embedded microcontrollers.

A systematic two-stage validation campaign was carried out to assess the performance margins of the synthesized control loop. First, a comprehensive numerical sensitivity analysis was conducted using a linear simulator identified from experimental results on the vehicle. This simulation phase characterized the coupled effects of the online adaptation gain $\Gamma_{\mathrm{NN}}$, the sigma-modification leakage factor α, and the hidden-layer size *N*, successfully mapping the hyperparameter space to optimize learning convergence speed while avoiding oscillatory transients. Crucially, the network weights were initialized from scratch without any prior offline pre-training, demonstrating true on-the-fly resilience.

The numerical findings were subsequently validated through real-time physical experimental tests using the Crazyflie 2.1+ micro-quadrotor platform constrained to an attitude tilting test bench. Under a persistent, highly asymmetrical unbalancing torque generated via physical payload displacement, the proposed DReLU-based augmented architecture successfully suppressed tracking offsets with minimal overshoot and negligible cross-channel dynamic coupling. When benchmarked against a literature-standard RBF adaptive network, the proposed scheme achieved a 52.6% reduction in embedded CPU execution core load while preserving comparable tracking accuracy metrics.

Future research will investigate the scalability of the DReLU adaptive framework to the outer loop by synthesizing an augmented position control layer. Additionally, further efforts will focus on evaluating network resilience under highly dynamic time-varying external moments, such as severe aerodynamic wind shear and complex ground effect interactions.

## Figures and Tables

**Table 1:** Nominal geometric, mass, and inertial properties of the Crazyflie 2.1+ micro-quadrotor utilized for numerical synthesis and experimental benchmarking.

Parameter	Symbol	Value
Total Mass	*m*	0.031kg
Rotor-to-CG Distance (*x*-axis)	*l*	0.049m
Rotor-to-CG Distance (*y*-axis)	*b*	0.049m
Inertia Tensor Diagonal	J	$\mathrm{diag}\left(1.0,1.0,3.0\right)\times 10^{-5}\;\mathrm{kg}\cdot m^{2}$

**Table 2:** Design parameters for the performance weighting functions $Q_{e}\left(s\right)$ of the inner (p,q) and outer (ϕ,θ) loops.

j	k	$\epsilon_{j}^{k}$	$M_{j}^{k}$	$\omega_{j}^{k,b}$
SAS	1	$10^{3}$	2π·1.0 rad/s	$10^{-2}$
SAS	2	$10^{3}$	2π·1.0 rad/s	$10^{-2}$
SAS	3	$10^{-2}$	$2\pi \cdot 10^{-2}$ rad/s	-
CAS	1	$10^{4}$	2π·1.0 rad/s	$10^{-3}$
CAS	2	$10^{4}$	2π·1.0 rad/s	$10^{-3}$
CAS	3	$10^{2}$	$2\pi \cdot 10^{-2}$ rad/s	-

**Table 3:** Design parameters for the control input $Q_{u}\left(s\right)$ weighting functions of the inner (p,q) and outer (ϕ,θ) loops.

j	k	$G_{u,j}^{k,0}$	$\omega_{u,j}^{k,c}$	$G_{u,j}^{k,1}$	$G_{u,j}^{k,\infty }$
SAS	1	100	2π·1.0 rad/s	1000	$10^{6}$
SAS	2	100	2π·1.0 rad/s	1000	$10^{6}$
SAS	3	0	2π·1.0 rad/s	-	1.0
CAS	1	0.01	2π·0.25 rad/s	0.1	1.0
CAS	2	0.01	2π·0.25 rad/s	0.1	1.0
CAS	3	-	-	-	

**Table 4:** Design parameters for the robustness $Q_{y}\left(s\right)$ weighting functions of the inner (p,q) and outer (ϕ,θ) loops.

j	k	$G_{y,j}^{k,0}$	$\omega_{y,j}^{k,c}$	$G_{y,j}^{k,1}$	$G_{y,j}^{k,\infty }$
SAS	1	0.2	2π·1.0 rad/s	2.0	50
SAS	2	0.2	2π·1.0 rad/s	2.0	50
SAS	3	-	-	-	
CAS	1	0.06	2π·1.0 rad/s	0.6	100
CAS	2	0.06	2π·1.0 rad/s	0.6	100
CAS	3	-	-	-	

**Table 5:** Discrete search grid for the adaptive learning rate $\Gamma_{\mathrm{NN}}$ and the sigma-modification parameter α evaluated during Sensitivity Analysis I.

Hyperparameter	Explored Set
$\Gamma_{\mathrm{NN}}$	$\left[1\times 10^{-4},\;5\times 10^{-4},\;1\times 10^{-3},\;5\times 10^{-3}\right]$
α	$\left[1\times 10^{-4},\;1\times 10^{-3},\;1\times 10^{-2},\;1\times 10^{-1}\right]$

**Table 6:** Quantitative performance metrics ($MAE_{\phi }$, $SD_{\phi }$) evaluated from 195 s to 220 s as a function of the adaptation rate $\Gamma_{\mathrm{NN}}$ under a constant leakage factor $\alpha =1\times 10^{-4}$.

$\Gamma_{\mathrm{NN}}$	${\mathrm{MAE}}_{\phi }$ [deg]	${\mathrm{SD}}_{\phi }$ [deg]	$\Delta_{\mathrm{TV}}$ [Nm]
$1\cdot 10^{-4}$	3.766	2.357	4.5424 $\cdot 10^{-6}$
$5\cdot 10^{-4}$	0.287	0.666	3.4211 $\cdot 10^{-6}$
$1\cdot 10^{-3}$	0.051	0.208	3.5573 $\cdot 10^{-6}$
$5\cdot 10^{-3}$	0.032	0.186	1.2178 $\cdot 10^{-5}$

**Table 7:** Quantitative performance metrics ($MAE_{\phi }$, $SD_{\phi }$) evaluated from 195 s to 220 s as a function of the leakage parameter α under a constant adaptation rate $\Gamma_{\mathrm{NN}}=1\times 10^{-3}$.

α	${\mathrm{MAE}}_{\phi }$ [deg]	${\mathrm{SD}}_{\phi }$ [deg]	$\Delta_{\mathrm{TV}}$ [Nm]
$1\cdot 10^{-4}$	0.051	0.208	3.557 $\cdot 10^{-6}$
$1\cdot 10^{-3}$	0.090	0.207	3.556 $\cdot 10^{-6}$
$1\cdot 10^{-2}$	0.470	0.204	3.556 $\cdot 10^{-6}$
$1\cdot 10^{-1}$	3.957	0.167	3.504 $\cdot 10^{-6}$

**Table 8:** Tracking accuracy metrics ($MAE_{\phi },SD_{\phi }$) evaluated from 195 s to 220 s across different hidden-layer dimensions (*N*) to determine the optimal onboard balance between modeling precision and computational burden.

Number of Neurons	${\mathrm{MAE}}_{\phi }$ [deg]	${\mathrm{SD}}_{\phi }$ [deg]	$\Delta_{\mathrm{TV}}$ [Nm]
5	0.420	0.856	3.5648 $\cdot 10^{-6}$
10	0.111	0.347	3.4402 $\cdot 10^{-6}$
15	0.051	0.208	3.5573 $\cdot 10^{-6}$
20	0.014	0.113	3.7627 $\cdot 10^{-6}$
25	0.002	0.086	3.9083 $\cdot 10^{-6}$
30	0.008	0.076	4.1426 $\cdot 10^{-6}$
35	0.013	0.080	4.3299 $\cdot 10^{-6}$

**Table 9:** Core parameterization and operational initialization profiles compiled for the real-time embedded implementation of the DReLU and benchmark RBF adaptive algorithms. Both architectures use the same number of neurons.

Parameter	Value
Neuron Count (*N*)	15
DReLU Learning Rate ($\Gamma_{\mathrm{DReLU}}$)	$1\times 10^{-3}$
DReLU Decay Factor ($\alpha_{\mathrm{DReLU}}$)	$1\times 10^{-4}$
DReLU Bias ($\zeta_{bias}$)	$\left[-\frac{\pi }{6}:\frac{\pi }{6}\right]$
RBF Learning Rate ($\Gamma_{\mathrm{RBF}}$)	5.0
RBF Decay Factor ($\alpha_{\mathrm{RBF}}$)	$1\times 10^{-3}$
Initial Weights (W(0))	$0_{(N+1)\times 3}$
RBF Centers (β)	$\left[-\frac{\pi }{6}:\frac{\pi }{6}\right]$
RBF Scaling Factor (σ)	0.08

**Table 10:** Onboard tracking performance indices and processing core footprints evaluated on the embedded STM32F405 microcontroller, highlighting the resource consumption efficiency of the proposed DReLU architecture over the RBF benchmark. The metrics in this table were evaluated for the last trapezoidal signal from 195 s to 220 s to avoid including the discontinuity of the doublets in the computation.

Activation Function	${\mathrm{MAE}}_{\phi }$ [deg]	${\mathrm{SD}}_{\phi }$ [deg]	CPU%	CPU% (Worst Case)
Proposed DReLU	1.114	1.020	1.464	1.710
Standard RBF	1.231	1.172	3.094	3.472

**Table 11:** Experimental results for θ and ψ angles, evaluated for the last trapezoidal signal from 195 s to 220 s.

Activation Function	${\mathrm{MAE}}_{\theta }$ [deg]	${\mathrm{SD}}_{\theta }$ [deg]	${\mathrm{MAE}}_{\psi }$ [deg]	${\mathrm{SD}}_{\psi }$ [deg]
Proposed DReLU	0.789	0.964	1.130	1.413
Standard RBF	1.405	1.717	2.523	2.807

## Data Availability

The raw data supporting the conclusions of this article will be made available by the authors on request.

## References

[1] de Oliveira Evald P.J.D., Aoki V.M., da Silva C.B., Cardoso D.S., Pinheiro P.M., da Costa Botelho S.S., Junior P.L.J.D. (2024). A Review on Quadrotor Attitude Control Strategies. Int. J. Intell. Robot. Appl..

[2] Hildmann H., Kovacs E. (2019). Review: Using Unmanned Aerial Vehicles (UAVs) as Mobile Sensing Platforms (MSPs) for Disaster Response, Civil Security and Public Safety. Drones.

[3] Nex F., Remondino F. (2014). UAV for 3D mapping applications: A review. Appl. Geomat..

[4] Gupta A., Fernando X. (2022). Simultaneous Localization and Mapping (SLAM) and Data Fusion in Unmanned Aerial Vehicles: Recent Advances and Challenges. Drones.

[5] Tagarakis A.C., Filippou E., Kalaitzidis D., Benos L., Busato P., Bochtis D. (2022). Proposing UGV and UAV Systems for 3D Mapping of Orchard Environments. Sensors.

[6] Ren H., Zhao Y., Xiao W., Hu Z. (2019). A review of UAV monitoring in mining areas: Current status and future perspectives. Int. J. Coal Sci. Technol..

[7] Malaver A., Motta N., Corke P., Gonzalez F. (2015). Development and Integration of a Solar Powered Unmanned Aerial Vehicle and a Wireless Sensor Network to Monitor Greenhouse Gases. Sensors.

[8] Camarillo-Escobedo R., Flores J.L., Marin-Montoya P., García-Torales G., Camarillo-Escobedo J.M. (2022). Smart Multi-Sensor System for Remote Air Quality Monitoring Using Unmanned Aerial Vehicle and LoRaWAN. Sensors.

[9] Mogili U.R., Deepak B.B.V.L. (2018). Review on Application of Drone Systems in Precision Agriculture. Procedia Comput. Sci..

[10] Zhang H., Wang L., Tian T., Yin J. (2021). A Review of Unmanned Aerial Vehicle Low-Altitude Remote Sensing (UAV-LARS) Use in Agricultural Monitoring in China. Remote Sens..

[11] Sassu A., Gambella F., Ghiani L., Mercenaro L., Caria M., Pazzona A.L. (2021). Advances in Unmanned Aerial System Remote Sensing for Precision Viticulture. Sensors.

[12] Jaimes A., Kota S., Gomez J. An approach to surveillance an area using swarm of fixed wing and quad-rotor unmanned aerial vehicles UAV (s). Proceedings of the IEEE International Conference on System of Systems Engineering.

[13] Sa I., Hrabar S., Corke P. (2015). Inspection of Pole-like Structures Using a Visual-Inertial Aided VTOL Platform with Shared Autonomy. Sensors.

[14] Rattanaamporn S., Perera A., Nguyen A., Ngo T.B., Chahl J. (2025). Drone Imaging and Sensors for Situational Awareness in Hazardous Environments: A Systematic Review. J. Sens. Actuator Netw..

[15] Hassani H., Mansouri A., Ahaitouf A. (2024). Robust Attitude Control of a Disturbed Multi-Rotor Aircraft: Experimental Comparisons. Proceedings of the International Conference on Digital Technologies and Applications.

[16] Lim H., Park J., Lee D., Kim H.J. (2012). Build your own quadrotor: Open-source projects on unmanned aerial vehicles. IEEE Robot. Autom. Mag..

[17] Goodarzi F., Lee D., Lee T. Geometric nonlinear PID control of a quadrotor UAV on SE (3). Proceedings of the European Control Conference (ECC).

[18] Liu S., Hou Z., Zheng J. Attitude adjustment of quadrotor aircraft platform via a data-driven model free adaptive control cascaded with intelligent PID. Proceedings of the Chinese Control and Decision Conference (CCDC).

[19] Jiao Q., Liu J., Zhang Y., Lian W. Analysis and design the controller for quadrotors based on PID control method. Proceedings of the 2018 33rd Youth Academic Annual Conference of Chinese Association of Automation (YAC).

[20] Meier L., Tanskanen P., Fraundorfer F., Pollefeys M. (2012). The pixhawk open-source computer vision framework for mavs. Int. Arch. Photogramm. Remote Sens. Spat. Inf. Sci..

[21] Candan F., Beke A., Kumbasar T. Design and Deployment of Fuzzy PID Controllers to the nano quadcopter Crazyflie 2.0. Proceedings of the 2018 Innovations in Intelligent Systems and Applications (INISTA).

[22] Lopez-Franco C., Gomez-Avila J., Alanis A.Y., Arana-Daniel N., Villaseñor C. (2017). Visual Servoing for an Autonomous Hexarotor Using a Neural Network Based PID Controller. Sensors.

[23] Andrade F.A.A., Guedes I.P., Carvalho G.F., Zachi A.R.L., Haddad D.B., Almeida L.F., de Melo A.G., Pinto M.F. (2022). Unmanned Aerial Vehicles Motion Control with Fuzzy Tuning of Cascaded-PID Gains. Machines.

[24] Wen S., Zhang Q., Deng J., Lan Y., Yin X., Shan J. (2018). Design and Experiment of a Variable Spray System for Unmanned Aerial Vehicles Based on PID and PWM Control. Appl. Sci..

[25] Hoffmann F., Goddemeier N., Bertram T. Attitude estimation and control of a quadrocopter. Proceedings of the 2010 IEEE/RSJ International Conference on Intelligent Robots and Systems.

[26] Bo G., Xin L., Hui Z., Ling W. Quadrotor helicopter attitude control using cascade PID. Proceedings of the 2016 Chinese Control and Decision Conference (CCDC).

[27] Panomrattanarug B., Higuchi K., Mora-Camino F. Attitude Control of a Quadrotor Aircraft Using LQR State Feedback Controller with Full Order State Observer. Proceedings of the The SICE Annual Conference 2013.

[28] Noordin A., Mohd Basri M.A., Mohamed Z., Mat Lazim I. (2021). Adaptive PID controller using sliding mode control approaches for quadrotor UAV attitude and position stabilization. Arab. J. Sci. Eng..

[29] Chen J., Long Y., Li T. (2024). Robust backstepping control for attitude tracking of a quadrotor based on integral linear extended state observer under input saturation. Nonlinear Dyn..

[30] Adigbli P., Grand C., Mouret J.B., Doncieux S. Nonlinear attitude and position control of a micro quadrotor using sliding mode and backstepping techniques. Proceedings of the 7th European Micro Air Vehicle Conference (MAV07).

[31] Izadi M., Shayan Z., Faieghi R. Multi-model predictive attitude control of quadrotors. Proceedings of the IEEE 20th International Conference on Automation Science and Engineering (CASE).

[32] D’Amato E., Tartaglione G., Blasi L., Mattei M. Nonlinear Dynamic Inversion with Neural Networks for the Flight Control of a Low Cost Tilt Rotor Uav. Proceedings of the 30th Congress of the International Council of the Aeronautical Sciences. International Council of the Aeronautical Sciences ICAS.

[33] Safwat E., Zhang W., Kamel A., Kassem M. (2020). Robustness analysis of modified incremental nonlinear dynamic inversion for small UAVs. Autom. Control Comput. Sci..

[34] Xie L., de Souza C.E. Robust *H*_∞_ control for linear systems with norm-bounded time-varying uncertainty. Proceedings of the 29th IEEE Conference on Decision and Control.

[35] Hui N., Guo Y., Han X., Wu B. (2024). Robust H-infinity dual cascade MPC-based attitude control study of a quadcopter UAV. Actuators.

[36] Hamza A., Mohamed A.H., El-Badawy A. Robust H-Infinity Control for a Quadrotor UAV. Proceedings of the AIAA Scitech Forum.

[37] Scordamaglia V., Sollazzo A., Mattei M. (2012). Fixed Structure Flight Control Design of an Over-Actuated Aircraft in the Presence of Actuators with Different Dynamic Performance. IFAC Proc. Vol..

[38] Jafar A., Fasih-UR-Rehman S., Fazal-UR-Rehman S., Ahmed N., Shehzad M.U. A robust *H*_∞_ control for unmanned aerial vehicle against atmospheric turbulence. Proceedings of the 2nd International Conference on Robotics and Artificial Intelligence (ICRAI).

[39] Noormohammadi-Asl A., Esrafilian O., Arzati M.A., Taghirad H.D. (2020). System Identification and H∞-Based Control of Quadrotor Attitude. Mech. Syst. Signal Process..

[40] Lavretsky E., Wise K.A. (2013). Robust and Adaptive Control with Aerospace Applications.

[41] Santos M.C.P., Rosales C.D., Sarapura J.A., Sarcinelli-Filho M., Carelli R. (2019). An adaptive dynamic controller for quadrotor to perform trajectory tracking tasks. J. Intell. Robot. Syst..

[42] Whitehead B., Bieniawski S. Model reference adaptive control of a quadrotor UAV. Proceedings of the AIAA Guidance, Navigation, and Control Conference.

[43] Whitaker H., Yamron J., Kezer A., M.I.T. Instrumentation Laboratory (1958). Design of Model Reference Adaptive Control Systems for Aircraft.

[44] Rothe J., Zevering J., Strohmeier M., Montenegro S. (2020). A Modified Model Reference Adaptive Controller (M-MRAC) Using an Updated MIT-rule for the Altitude of a UAV. Electronics.

[45] Khalil A., Jaradat M.A., Mukhopadhyay S., Abdel-Hafez M.F. (2023). Autonomous Control of a Hybrid Rolling and Flying Caged Drone for Leak Detection in HVAC Ducts. IEEE/ASME Trans. Mechatron..

[46] Ale Isaac M.S., Luna M.A., Ragab A.R., Ale Eshagh Khoeini M.M., Kalra R., Campoy P., Flores Peña P., Molina M. (2022). Medium-Scale Uavs: A Practical Control System Considering Aerodynamics Analysis. Drones.

[47] Nguyen N.T. (2018). Model-reference adaptive control. Model-Reference Adaptive Control: A Primer.

[48] Anderson R.B., Marshall J.A., L’Afflitto A. (2021). Novel Model Reference Adaptive Control Laws for Improved Transient Dynamics and Guaranteed Saturation Constraints. J. Frankl. Inst..

[49] Lavretsky E., Wise K.A. (2024). Robust adaptive control. Robust and Adaptive Control: With Aerospace Applications.

[50] Lara D., Romero G., Ibarra R., Sánchez A., Pegard C. Onboard System for Flight Control of a Small UAV. Proceedings of the World Automation Congress 2012.

[51] Zhang D., Wei B. (2016). Convergence Performance Comparisons of PID, MRAC, and PID+ MRAC Hybrid Controller. Front. Mech. Eng..

[52] Li M., Cai Z., Zhao J., Wang J., Wang Y. (2022). Disturbance Rejection and High Dynamic Quadrotor Control Based on Reinforcement Learning and Supervised Learning. Neural Comput. Appl..

[53] Orsag M., Korpela C.M., Bogdan S., Oh P.Y. (2014). Hybrid adaptive control for aerial manipulation. J. Intell. Robot. Syst..

[54] Yang X., Hu X., Ye H., Liu W., Shen H. (2023). Fraction-Order MRAC Method Based Fault Tolerant Control for Plant Protection UAV with Actuator Failure and Uncertainty. Int. J. Control. Autom. Syst..

[55] Tanveer A., Afzaal N., Murawwat S., Aleem S., Sayeda F. Mitigating Flight Performance Errors in UAVs through Hybrid MRAC controller. Proceedings of the International Conference on Emerging Trends in Electrical, Control, and Telecommunication Engineering (ETECTE).

[56] Li Q., Tong S., Chai T. *H*_∞_ tracking of unknown nonlinear systems using neural network. Proceedings of the 12th IEEE International Symposium on Intelligent Control.

[57] Rovithakis G. Reinforcing robustness using high order neural network controllers. Proceedings of the 37th IEEE Conference on Decision and Control.

[58] Yang Y.S., Wang X.F. (2007). Adaptive *H*_∞_ tracking control for a class of uncertain nonlinear systems using radial-basis-function neural networks. Neurocomputing.

[59] Li Y., Sundararajan N., Saratchandran P., Wang Z. (2004). Robust neuro-*H*_∞_ controller design for aircraft auto-landing. IEEE Trans. Aerosp. Electron. Syst..

[60] Nicol C., Macnab C., Ramirez-Serrano A. Robust neural network control of a quadrotor helicopter. Proceedings of the Canadian Conference on Electrical and Computer Engineering.

[61] Lin C.K., Wang S.D. (2004). An adaptive *H*_∞_ controller design for bank-to-turn missiles using ridge Gaussian neural networks. IEEE Trans. Neural Netw..

[62] Bassolillo S.R., Raspaolo G., Blasi L., D’Amato E., Notaro I. Neural Network Based Model Reference Adaptive Attitude Control for a Micro Unmanned Air Vehicle. Proceedings of the 11th International Conference on Control, Decision and Information Technologies (CoDIT).

[63] Madani T., Benallegue A. (2008). Adaptive control via backstepping technique and neural networks of a quadrotor helicopter. IFAC Proc. Vol..

[64] Shastry A.K., Kothari M., Abhishek A. Development of flight dynamics model of quadrotor. Proceedings of the AIAA Guidance, Navigation, and Control Conference.

[65] Chaudhuri B., Pal B.C., Zolotas A.C., Jaimoukha I.M., Green T.C. (2003). Mixed-Sensitivity Approach to *H*_∞_ Control of Power System Oscillations Employing Multiple FACTS Devices. IEEE Trans. Power Syst..

[66] Humaidi A., Hameed A. (2017). Robust MRAC for a wing rock phenomenon in delta wing aircrafts. AUT J. Model. Simul..

[67] Jurado F., Ollervides-Vazquez E.J. (2025). Decentralized Robust Direct MRAC via e-Modification for the Pose of a Quadrotor UAV. Appl. Sci..

[68] Ioannou P., Sun J. (2014). Robust Adaptive Control.

[69] Chicco D., Warrens M.J., Jurman G. (2021). The Coefficient of Determination R-squared Is More Informative than SMAPE, MAE, MAPE, MSE and RMSE in Regression Analysis Evaluation. PeerJ Comput. Sci..

[70] Klán P., Gorez R. (2008). PI Controller Design for Actuator Preservation. IFAC Proc. Vol..

[71] Macêdo D., Zanchettin C., Oliveira A.L., Ludermir T. (2019). Enhancing Batch Normalized Convolutional Networks Using Displaced Rectifier Linear Units: A Systematic Comparative Study. Expert Syst. Appl..

[72] Slama S., Errachdi A., Benrejeb M. (2019). Online MRAC Method Using Neural Networks Based on Variable Learning Rate for Non-Linear Systems. Int. J. Comput. Appl. Technol..

[73] Liu M., Dong X., Li Q., Ren Z. Model Reference Adaptive Control of a Quadrotor UAV based on RBF Neural Networks. Proceedings of the IEEE CSAA Guidance, Navigation and Control Conference (CGNCC).

[74] Chen W., Ding Y., Weng F., Liang C., Li J. (2025). Global fast terminal fuzzy sliding mode control of quadrotor UAV based on RBF neural network. Sensors.

